# Dwarf mongooses pre-emptively alter their behaviour relative to the threat posed by different rival groups

**DOI:** 10.1038/s41559-026-03104-3

**Published:** 2026-06-16

**Authors:** Josh J. Arbon, Amy Morris-Drake, Julie M. Kern, Andrew N. Radford

**Affiliations:** School of Biological Sciences, University of Bristol, Bristol, UK

**Keywords:** Behavioural ecology, Social evolution, Animal behaviour

## Abstract

Intergroup conflict is a potent evolutionary force across taxa, but little research has investigated how social animals pre-emptively change their behaviour in scenarios where contests with rivals are more likely. Moreover, the few studies examining this aspect of intergroup conflict fail to consider rival characteristics despite them determining contest outcomes and interaction costs, which define the threat level different groups pose. Here we show how non-human animals can tailor their anticipatory behaviour to the specific threat posed by rivals, using 10 years of detailed behavioural observations and GPS data. We demonstrate that dwarf mongooses (*Helogale parvula*) adjust their space use, information provisioning and resource defence dependent on the relative group size of neighbours to help them mitigate the threat from both well-matched competitors and more dangerous larger rivals. By contrast, behavioural differences between the core and edge of home ranges were more equivocal, highlighting the importance of considering rival characteristics rather than just spatial location as an indicator of threat level. Our results showcase how animals have evolved to be best prepared for a key part of their ecology, potential future contests, indicating abilities that allow them to survive and thrive in a landscape of intergroup conflict.

## Abstract (web summary)

Using 10 years of GPS data and behavioural observations, the authors show that wild dwarf mongooses pre-emptively alter their behaviours, including space use and sentinel calling, in relation to the level of threat posed by the size of rival groups.

## Main

Intergroup conflict is a potent evolutionary force that has shaped social structure, territoriality and cooperation from ants to apes, including in humans^1–6^. Contests between conspecific groups have been well-studied in diverse taxa, highlighting a range of immediate and delayed fitness consequences^1,2^. Antagonistic interactions with outsiders, especially those involving physical attacks, can lead to loss of life or breeding position^7,8^, while there can also be knock-on consequences from contest-induced injuries and takeovers, including changes in behaviour and space use^9–12^. The costly nature of intergroup contests means that, much as information about predator occurrence in the environment generates a ‘landscape of fear’ for prey to navigate^13,14^, the presence of conspecific rivals creates a ‘landscape of conflict’. In line with the way that prey pre-emptively alter their behaviour in relation to the ecological pressure of the predation landscape (that is, use experience and current information to optimize behaviour ahead of a potential future interaction), animal groups should benefit by responding to the variation in threat posed by rival groups^15,16^.

Humans respond to the threat of intergroup contests by increasing their surveillance for rivals when in conflict zones and moving quietly through enemy territory to avoid detection^17,18^. There are also a few examples of similar location-related behaviour in non-human animals. For instance, recent work showed that two chimpanzee (*Pan troglodytes*) groups used elevated vantage points when moving towards territorial borders^16^, while raiding parties of males go silent when entering another territory^19^. However, research has been largely primate focused, and the evidence is equivocal: there is inconsistency between species in how space use and behaviour relates to the increase in intergroup risk that is predicted towards territory borders^14,20,21^. One reason is that the location-based risk of encountering other groups often correlates with environmental variables, such as food availability^21^ or habitat type^22^, generating edge effects that can mask the threat attributable to intergroup rivals. Moreover, there is large variation in the threat posed by different rival groups; this key factor is currently unexplored in a pre-emptive context.

In contrast to predators, where each individual of a given species provides a relatively comparable threat, conspecific groups can vary hugely in their competitive ability, with the intensity and outcome of intergroup contests known to depend on the characteristics of the rivals^23–28^. Arguably the most important single factor is group size, which relates positively to resource-holding potential in many social species^24,25,29^. As larger groups are usually at an advantage in contests^23–25^, engaging with them carries greater risks, including potential mortality; in many cases, early detection and retreat will be the best strategy for relatively smaller groups^30,31^. However, well-matched competitors (for example, those with similar group sizes) often participate in more prolonged, escalated contests, which incur greater costs in terms of time, energy and injury risk^26,27^; preparing for such encounters may give groups an important advantage and be just as important as preparing for much large rivals. However, it is unknown in what ways the relative group size of rivals influences the pre-emptive decisions of non-human animals and how they integrate variable threat levels into their ecological landscape of conflict.

Here we combine 10 years of global positioning system (GPS) data and behavioural observations on wild dwarf mongooses (*Helogale parvula*) to investigate how groups change their pre-emptive behaviour relative to the specific threat posed by intergroup rivals. We consider how the pre-emptive behaviour of mongoose groups is affected by the relative group size of neighbours, predicting that groups would adjust their space use, information gathering and resource defence to help them mitigate the threat from both well-matched competitors and more dangerous (that is, larger) rivals. We also examine the same behaviours in home range core areas versus edge areas, predicting that behavioural differences commensurate with a greater perceived threat in edge areas as these are at the borders with other groups.

## Results

We mapped the space used by 12 wild, habituated dwarf mongoose groups over 69 group-years (maximum 8 groups monitored at any one time), and analysed over 1,600 daily movement tracks, 16,000 sentinel (raised guarding) observations and 2,000 sleeping burrow returns, to investigate differences in pre-emptive behaviour depending on the intergroup threat level. Dwarf mongooses are territorial mammals of Southern Africa^32,33^, regularly participating in intergroup interactions (IGIs) with neighbours that can result in injury and even mortality^34^. Crucially, we investigate periods occurring outside the direct context of an IGI to assess pre-emptive behaviour; the vast majority of data come from days without IGIs—98% of 2,941 days—and for the small proportion of data from days where IGIs occurred (*N* = 59 days), only data from at least 30 min before the first interaction of the day were included.

### Effects of relative group size

**Fig. 1:**
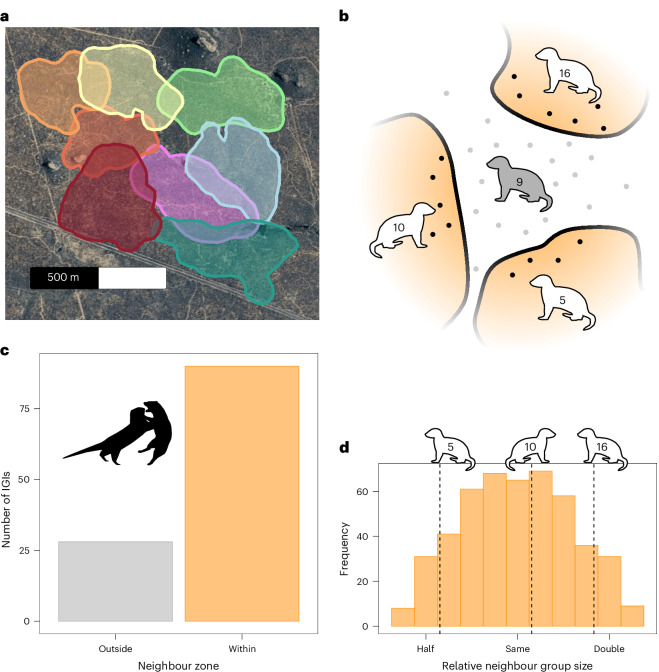
Neighbour zones carry an intergroup threat but of varying level. **a**, Home ranges (95% UDs) of the eight groups in the study population during 2018, highlighting both exclusive and overlapping areas of space use. **b**, When analysing the effect of relative neighbour group size, data from a given focal group (grey silhouette, group size of nine) were considered when they occurred within the home range of a neighbour, termed a neighbour zone (white silhouettes, black points). Data falling outside of neighbour zones (grey points) were not considered. **c**, The number of observed IGIs that fell within and outside neighbour zones (*N* = 118 IGIs). **d**, The distribution of relative neighbour group sizes from data aggregated at the month level. Dashed lines highlight the relative group size of neighbours in **c** (5, 10 and 16 adults) relative to the focal group (9 adults). Imagery in **a** from Google, ©2026 Airbus, CNES/Airbus, Maxar Technologies.

We first assessed the importance of relative neighbour group size (number of adults; aged 1 year and older), analysing behaviours occurring in ‘neighbour zones’. We used GPS data to generate 95% utilization distributions (UDs) of study groups (Fig. 1a). For each behavioural observation in our datasets, we assessed whether its location fell within the 95% UD of a neighbour (Fig. 1b, orange areas; as calculated from the previous 90 days, a balance between data availability and recency); hereafter ‘neighbour zone’. More than three-quarters of IGIs occurred in neighbour zones (Fig. 1c), highlighting the escalated risk of encounter when in these areas. Using sections of movement tracks, sentinel observations and returns to an evening burrow that fell within a neighbour zone, we assessed how the relative group sizes of the focal and neighbour group affected behaviour of the former. Groups had a mean of 7.9 adults, with a mean relative group-size ratio of 1:1.4 between pairs of neighbours (Fig. 1d); groups often had larger and smaller neighbours simultaneously (as in Fig. 1b), but relative group size was not significantly related to the size of neighbour zones (*P* = 0.839; Supplementary Table 1). We modelled both a linear term and a quadratic term for relative neighbour group size to assess whether behavioural changes were greatest in response to the threat from larger (and therefore more competitive—linear) or similarly sized groups (with whom contests may be most likely to escalate—quadratic). Unless stated, the quadratic effect was dropped (as nonsignificant) to enable reporting of the linear term.

**Fig. 2:**
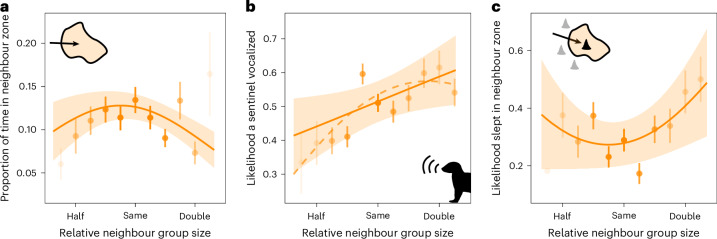
The relative size of neighbouring groups affects multiple mongoose behaviours. **a**–**c**, The effect of relative neighbour group size on the proportion of time spent inside a neighbour zone (*N* = 511 months with at least five tracked days) (**a**), the likelihood that a given sentinel bout was announced vocally (*N* = 1,936 bouts) (**b**) and the likelihood that a group slept in the neighbour zone on days they entered it (*N* = 893 days) (**c**). Lines and shading are model-estimated marginal means and 95% CIs. The dashed line in **b** represents a nonsignificant quadratic trend. Points and arms are illustratively discretized raw data means and standard errors, and in **b** are adjusted for the marginal effect of absolute group size (Methods and Supplementary Fig. 1). Opacity of points/arms represents data density, with darker points corresponding to more observations.

When in a neighbour zone, the relative group size of that neighbour affected multiple aspects of focal group behaviour. While there was no significant effect on the likelihood of entering a neighbour zone (*P* = 0.274; Supplementary Table 2a), relative group size did affect the proportion of time spent by the focal group in the neighbour zone. When accounting for the size of home range overlap between neighbour pairs (Supplementary Table 2b), groups spent more time in the neighbour zones of similarly sized and slightly smaller neighbours, and less time in the zones of much larger neighbours (quadratic term: *P* = 0.003; Fig. 2a). When groups were inside neighbour zones, relative neighbour group size affected both sentinel behaviour and burrow use but not movement characteristics (speed: *P* = 0.470; area covered: *P* = 0.839; Supplementary Table 3). When foraging, mongoose groups exhibit a coordinated vigilance system known as sentinel behaviour to look for both predators and conspecifics^35,36^. While there was no significant effect of relative neighbour group size on sentinel effort (likelihood of sentinel presence: *P* = 0.171; duration of bouts: *P* = 0.514; Supplementary Table 4a,b), relative neighbour group size did affect the likelihood that sentinels announced their presence to groupmates by giving ‘surveillance’ calls^37^. There was a nonsignificant trend for a quadratic effect of relative neighbour group size on sentinel vocalizations (*P* = 0.053; Fig. 2b, dashed line, and Supplementary Table 4c); when this quadratic term was removed, there was a significant linear increase in likelihood of vocalization (*P* = 0.042), with sentinels 8.2% more likely to announce their presence for each doubling of relative neighbour group size (95% confidence interval (CI) 0.1–13.1%; solid line, Fig. 2b). Dwarf mongooses are strictly diurnal and rely on underground burrows for overnight refuge^38^; although these are distributed throughout the landscape (Supplementary Fig. 2), around one-quarter of all IGIs occur at burrows, and more than 5% of burrows are used by multiple groups on different nights (Dwarf Mongoose Research Project (DMRP), unpublished data). On days when groups entered a neighbour zone, relative neighbour group size affected whether the group slept within the neighbour zone (quadratic term: *P* = 0.028; Supplementary Table 5a). Groups were least likely to sleep in the neighbour zone of well-matched competitors than those much smaller or larger than them (Fig. 2c). There was also a nonsignificant trend for groups to return to the burrow later relative to sunset with increasing relative neighbour group size (*P* = 0.098; Supplementary Table 5b).

### Effects of home range location

**Fig. 3:**
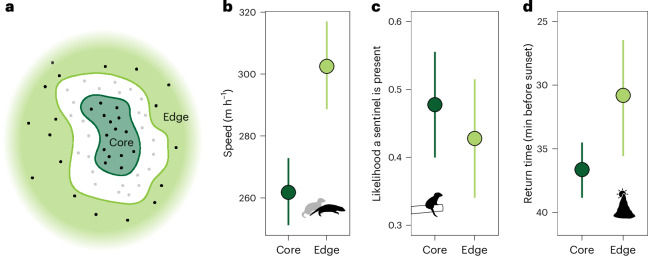
Location in a home range affects mongoose behaviours. **a**, A schematic of core and edge area assignment. Core areas were designated as those within the 50% UD (dark) and edge areas those outside of the 75% UD (light). Data falling in the buffer area between (grey points in white area) were not analysed. **b**–**d**, The model-estimated marginal means and 95% CIs of movement speed (*N* = 1,653 days) (**b**), the likelihood that a sentinel was present in a given scan sample (*N* = 16,660 scan samples) (**c**) and return time to the burrow in minutes before sunset (**d**), such that higher on the *y* axis is closer to sunset (*N* = 2,013 evenings).

We then investigated the same behaviours relative to the location within a focal group’s home range, with the implicit assumption that intergroup threat is greater in edge than core areas. We took all behaviours that fell within the 50% UD (core; Fig. 3a) and compared them with all behaviours occurring outside the 75% UD (edge). Groups moved 17.6% faster (11.9–23.2%; *P* < 0.001; Fig. 3b and Supplementary Table 6a) and covered 39.8% more unique area per unit time (29.2–50.4%; *P* = 0.002; Supplementary Table 6b) when in edge areas compared with core areas. When in edge areas, a sentinel was present in 5.5% (2.5–8.1%) fewer scan samples (*P* = 0.002; Fig. 3c and Supplementary Table 7a), and sentinel bouts were 15.7% (9.5–22.9%) shorter in duration (*P* < 0.001; Supplementary Table 7b) relative to core areas, although the location within the home range had no significant effect on whether sentinels vocalized to announce their presence (*P* = 0.290; Supplementary Table 7c). Groups also returned to their burrows 13.1% (3.3–23.2%) later relative to sunset when the burrow was in an edge area relative to when it was in a core area (*P* = 0.015; Fig. 3d and Supplementary Table 8). The effects of location on behaviour were qualitatively the same if sentinel and burrow observations were assigned to 5% UDs from 50–100% and modelled with location as a continuous variable (‘location-based behaviour’ in Supplementary Analysis).

## Discussion

Our work demonstrates that dwarf mongooses pre-emptively alter a suite of behaviours in relation to intergroup threat level. Groups spent more time in areas shared with more evenly matched rivals but were more likely to retreat from these areas to sleep when competitors were similar in group size. Mongoose groups also altered their behaviour to minimize the potential costs of interactions with those larger in group size than them by increasing within-group communication. By contrast, we found mixed evidence that behaviour differs between core and edge locations in a manner consistent with the likelihood of intergroup encounter. We provide evidence that the anticipatory behaviour of non-human animal groups varies with the characteristics of, and thus level of threat posed by, different rivals. These findings highlight an important approach for understanding the dangers of intergroup conflict at the landscape scale.

We found evidence that groups pre-emptively adjust their behaviour on the basis of the specific threat posed by different rival groups, in line with what is known about how relative group sizes affect contests^24–26^. For instance, the likelihood of sentinels vocalizing increased with relative neighbour group size. Greater vocal announcement of sentinel bouts highlights a need to provide group members with up-to-date social information when threat is high, aiding group coordination and enabling adaptive reductions in vigilance by foragers^37,39^, even at the cost of increasing detectability to potential predators. As nearly half of dwarf mongoose IGIs escalate to physical fights^34^, and as multiple group members can attack a single rival individual simultaneously, smaller groups are clearly disadvantaged when faced with larger counterparts. Thus, there are potential adaptive benefits of sentinels increasing communication when the rival is larger. By calling when being a sentinel, individuals providing this valuable service in a high-risk scenario may also be more likely to have their investment rewarded through grooming^40^, an idea that could be tested experimentally in future work^40,41^.

Other behavioural changes showed a quadratic relationship with relative neighbour group size, indicating how preparing for well-matched neighbours can be equally important. The change in the proportion of time spent in the neighbour zone reflects not only differences in the need to defend border areas by occupying contested space and directly repelling rivals (that is, by engaging in IGIs) but also by more thoroughly investigating and depositing scent marks^34,36^. When mismatches in group size are great, smaller groups may not be able to risk prolonged exposure to large rivals, while those larger groups need not defend a border so vigorously if they can more easily repel rivals and take ownership of an area. Failure to maintain a border presence against similarly competitive groups, however, may lead to territorial incursions that cannot easily be reversed. We also saw changes in burrow-selection behaviour relative to neighbour group size. Reductions in sleeping in neighbour zones could represent a mitigation against costly conflict at burrows: while burrows are key resources, around one-quarter of all IGIs occur at burrows (DMRP, unpublished data), and as burrows are often only accessible by one or a few entrances, escalation in conflict in these locations may be especially costly. Groups may instead be retreating to sleep in locations that they perceive to be less risky when the likelihood of an escalated conflict is higher. These changes in behaviour may also be influenced by unmeasured variables such as habitat quality; while the overlap between groups was not related to their relative group size, it is unknown how the quality of these areas is perceived by the study groups. Collectively, these results indicate that, while larger groups have the potential to be more dangerous, the increased risk of escalation and lesser certainty in contest outcome when encountering well-matched competitors^24,26^ may make the latter threat just as important to mitigate with pre-emptive behaviour.

We also found evidence that mongooses differ in their behaviour relative to the location within their home range, but the observed differences are not consistently in line with adaptive responses to intergroup threat. As suggested by the relative group-size analyses, we found that mongooses returned later to burrows when the intergroup threat level was higher (in home range edge areas in this case). However, the sentinel findings were opposite to the increase in effort at edges that we would predict if groups were watching out more for neighbours. Mongooses might be reluctant to place themselves in an elevated position where they could be easily detected by rivals, but edge effects provide a likely alternative explanation. If groups forage less successfully in peripheral areas—owing to increased personal vigilance or lesser resource abundance—we may see less sentinel activity there than in core areas as it is a state-dependent behaviour^42^. We did observe faster movement speeds of groups when in home range edges, which could represent efforts to reduce time in areas associated with intergroup risk or conversely reflect search behaviour of more aggressive groups. However, it is also likely that there are other behavioural and environmental differences between core and edge that will affect movement behaviour. For instance, groups need to lay down fresh scent marks at latrine sites that are found across home range edges^43,44^, which may require groups to cover large distances quickly, without any need to evaluate the potential risk of encountering neighbours. In addition, other variables such as food availability or familiarity with safe refuges may affect movement characteristics^45,46^. Combined with an absence of a relative group-size effect on movement characteristics and sentinel effort, we remain cautious about interpreting these location-based differences in movement as being solely driven by intergroup risk. This set of results, along with mixed location-related findings in previous studies^14,20–22^, highlights the potential difficulty in interpreting spatial position as a sole proxy of intergroup threat and emphasises the benefit of considering the different threat posed by rivals with different characteristics (for example, group size).

Pre-emptive behavioural changes in relation to intergroup threat have the potential to be a more pervasive consequence of a territorial life-history than contests alone. Much as the threat of predation alters the behaviour of prey animals outside of direct interactions^13^, with impacts on population- and community-level processes^47,48^, the need to gather information and pre-emptively change behaviour relative to conspecific groups adds an important layer to landscapes of conflict. Behavioural changes tailored to the threat presented by particular potential rivals may help to explain how groups in many species appear to share home range areas without suffering excess aggression and the associated mortality risk^14,21^. However, these behavioural changes are unlikely to be cost-free and will be traded off against optimum actions in other contexts. For example, spending disproportionate amounts of time occupying certain areas could result in resource depletion and reductions in foraging success. Some behaviours may be more constrained and less easily altered over large timescales, and only show changes when threat reaches a certain likelihood—for instance, when there has been repeated information indicating imminent rival presence^49^. In such scenarios, greater information gathering could also aid in detection of predators^34^, highlighting how some behaviours can be synergistic and therefore may evolve concurrently. Increasing our understanding of how animal groups alter their day-to-day behaviour to counter the threat of rivals will therefore help us to evaluate the trade-offs individuals constantly make to balance key needs.

An outstanding question is how animals, including dwarf mongooses, assess intergroup threat when there is no current direct contact with rivals. One possibility is information from secondary cues in the environment. As with many territorial mammals, mongooses are habitual scent markers^44^, and experimental work has demonstrated immediate behavioural change in mongooses following the presentation of faecal samples^33,36,49^. However, as only 60% ± 25% (mean ± s.d.) of adult group members deposit scent at a given latrine (DMRP, unpublished data), this information would at best represent a coarse approximation of neighbour group size. Another non-mutually exclusive explanation for pre-emptive behaviour is that mongooses use memories of previous encounters, both of interactions and neighbour’s secondary cues, to modulate their behaviour. Dwarf mongoose group sizes and home ranges are relatively stable^32,38,50^, and therefore information from an encounter with a neighbour will probably be accurate in the following days and weeks, whereas this may not be true for species that exhibit other social structures such as fission–fusion societies^51^. A requirement to remember locations and encounters with rivals has been argued to be a driver of cognitive evolution^16,52^, but in animal societies where information is reliable over time, this is not necessarily highly cognitively demanding. A better understanding of the information animals need to gather, process and retain is required for a robust appraisal of the cognitive challenge that intergroup conflict presents. Recent methodological advances are beginning to unpick the neurological bases of social interactions across space^53^; such experiments that can disentangle the responses to direct and indirect cues of rival presence from spatially anchored memories of outsider interactions will help further our evolutionary understanding of pre-emptive behavioural changes in the context of intergroup conflict and the underpinning cognitive apparatus^52^.

Our results reveal how dwarf mongooses can pre-emptively change their behaviour relative to the intergroup threat level, an ability that we expect to be widespread across the diverse taxa with life-histories shaped by intergroup conflict. In this study, we have investigated behavioural changes in relation to relative group size, but the threat presented by rival groups in other species can also vary depending on sex ratio^23^, relatedness between groups^28^ or fluctuations in motivation^29,54^ and the environment^55^. While our focus was on space use, movement, sentinelling and burrow use, for which we had large sample sizes, other behaviours such as scent-marking and intragroup affiliation may also provide important in a pre-emptive context. Studying groups outside of direct contests will provide insight into an animal’s view of its landscape of conflict, revealing nuances in behaviour that affect many aspects of daily life seemingly far-removed from fighting rivals. To understand fully the pervasive impacts of intergroup conflict, we must study the complete timeline of behaviours—not just those during and after contests but also those that occur in anticipation.

## Methods

### Study species

Dwarf mongooses are sexually monomorphic, group-living, territorial mammals found throughout Southern Africa^32,50^. They live in cooperatively breeding groups comprising a dominant breeding pair, their offspring and immigrants, with an overall balanced sex ratio^32^. Both sexes disperse and immigrate into new groups resulting in a relatively even relatedness structure between sexes^32^. Members of a group move cohesively in the environment; larger groups travel further daily distances and cover more area, ultimately resulting in larger groups occupying larger areas^38^. Groups routinely deposit scent marks at regularly used latrine sites, which fall predominantly near home range boundaries^36,43^. While groups do not patrol boundaries in the same way as some other species^56,57^, they regularly show directed movement to these marking locations, and the same latrine sites have been used by pairs of neighbouring groups over multiple years (DMRP, unpublished data). Groups engage directly with neighbours when they are encountered; these events are termed IGIs^33,36,38^. When IGIs occur, both male and female adults participate at equivalent rates, and the majority of IGIs result in agonistic physical interactions between the groups, occasionally resulting in injury and even mortality^34^; unlike some species, IGIs very rarely involve affiliative behaviour between members of different groups, and there is no evidence for extra-group paternity as seen in closely related species^6,58^. Groups may use indirect cues (for example, scent marks) and memories of past interactions to assess neighbour group size and the likelihood of encounter, even when there are no direct indicators that another group is currently nearby^43,59^.

In addition to the threat posed by neighbouring conspecific groups, dwarf mongooses are also depredated by a variety of diurnal and crepuscular predators, including many mammals and birds of prey^35^. As an adaptation to such a predator-rich landscape, dwarf mongooses perform coordinated vigilance known as sentinel behaviour, where one individual climbs to an elevated position and watches for danger^35,60^. During their bout, sentinels often announce their presence to the group by producing vocalizations termed surveillance calls, enabling foragers to reduce their vigilance^37,39^. Sentinel behaviour increases in response to the simulation of intergroup intrusions^36,49^, suggesting that sentinels can also act to detect potentially threatening conspecifics as well as heterospecifics. Dwarf mongooses are strictly diurnal and rely on burrows, mostly excavated termite mounds, in which to spend the night and avoid crepuscular and nocturnal predators^38,61^. Groups routinely switch burrows^38^, and burrows are found evenly distributed throughout the habitat (Supplementary Fig. 2).

### Ethics

Work was conducted under permission from the Limpopo Department of Economic Development, Environment and Tourism (permit no. 001CPM403-00013). Ethical approval was granted from the University of Pretoria, South Africa (Animal Ethics Committee: NAS321/2022) and the University of Bristol, UK (Animal Welfare and Ethics Review Body: UB/11/038), and the study was performed in line with The Association for the Study of Animal Behaviour guidelines for the ethical treatment of animals^62^.

### Data collection

All data were collected at Sorabi Rock Lodge, Limpopo Province, South Africa (24.211° S, 30.775° E), as part of the long-term Dwarf Mongoose Research Project. Data were collected between September 2013 and September 2023 from 12 groups of wild, habituated dwarf mongooses. During the winter months (~May–September), groups were followed for the entire day, from when they first emerged in the morning from the overnight sleeping burrow until they returned and went into a sleeping burrow in the evening. During the hot summer months (~October–April), groups were followed for ~3 h from when they left the overnight sleeping burrow, at which point the observer would leave the group during the heat of the day, returning to follow the group for 2–3 h before they returned to a sleeping burrow in the evening. This summer pattern of data collection was facilitated by mongooses becoming inactive in the heat of the day, often retreating below ground at a day refuge until later in the afternoon when it became cooler. Groups were observed for a mean of 9.2 days per month over the study period, usually in 2–3-day blocks^38^.

Observers collected three core datasets, which we use in this study: life-history, movement and behaviour data. The identity of all present group members was recorded during every observation session, enabled by the periodic application of unique blonde hair-dye marks for individual recognition. Birth data were collected to enable accurate designation of adult group members (individuals >1 year old) and therefore calculate adult group sizes. To generate a group size for neighbours on days when we did not visit a group, we used linear interpolation from the observed group sizes on either side of the unknown date. Observers continually recorded location data by positioning themselves close to the centroid of the group, taking the group track on a Garmin Etrex 10 (Garmin) handheld GPS device recording fixes typically at <3 m of accuracy^38^. We use these data to generate UDs and movement metrics, and to infer the locations of spatially anchored behavioural observations (that is, sentinel bouts and burrow uses).

Multiple behaviours were recorded during observation sessions. All observed IGIs were noted, and their location was logged with GPS. Sentinel behaviour was recorded through a combination of routine scan samples and ad libitum observations^35,40^. Scan samples were conducted every 30 min to assess whether a sentinel was present. At all times, other sentinel bouts were recorded with more detailed information on the duration of the bout and whether the sentinel announced its presence through vocalizations^37^; for both duration and vocalization data, only sentinel bouts where the observer witnessed the full bout were used for analysis. The location of sentinel scan samples and ad libitum bouts was inferred from daily GPS tracks. Sentinel data were assigned a location on the basis of the closest recorded fix in time within a cut-off of 2 min—any sentinel data without a corresponding location within 2 min were not analysed. Return time to the evening sleeping burrow was recorded as the time that 50% of the adult group members returned to the burrow that they subsequently went to sleep in. The location of all known burrows where timing data were obtained was logged with GPS. Over the study period, we observed 728 unique sleeping locations being used by mongoose groups (Supplementary Fig. 2).

### Data processing and analysis

All processing and analyses were conducted in R^63^ (version 4.5.1). First, we subsampled all GPS data to a target rate of one fix per minute to generate a standardized sampling rate, using the method outlined in ref. ^64^. Throughout, we calculated UDs using the most recent 90 days of GPS data for those groups with a minimum of 20 days of data available (as in ref. ^38^), using functions from the R package ‘adehabitatHR’ ^65^ (version 0.4.22). A period of 90 days was chosen as a compromise between capturing enough data to generate meaningful UDs while keeping assessed areas recent enough for the specific aims of the study. When generating UDs, we chose to use ‘standard’ (not autocorrelation corrected) kernel density estimation (KDE) to ensure our estimates of where mongoose groups had moved in the previous 90 days was conservative. When analysing movement data from our study system, we have previously found that estimating space use using autocorrelation-corrected KDE methods has resulted in estimation of home range areas ~1.4× larger than those estimated using KDE methods, including areas we have never seen groups over many years of observation^38^. This reduced the estimated used space (and therefore number of included behavioural data points carried into analysis) relative to calculating overlaps using an autocorrelation-corrected KDE method (implemented through continuous time movement models^66^). However, it ensured higher confidence in our assessment that a certain location was observed to be within the space recently used by a neighbour.

To assess the effects of relative neighbour group size on behaviour, we took each day with a recorded behavioural observation (movement tracking, sentinel observation and/or burrow use in the evening) and isolated those for each focal group that fell within the 95% UD of a neighbour (Fig. 1b), termed the neighbour zone, using functions from the R package ‘sf’^67^ (version 1.0–24). For these behavioural data in neighbour zones, we calculated the relative neighbour group size for each identified neighbour as the base 10 logarithm of neighbour group size/focal group size, such that relative group-size differences were centred on, and symmetrical around, 0 (for example, |log_10_(2)| = |log_10_(1/2)|; Fig. 1d). Models investigating the effect of relative neighbour group size (Supplementary Tables 2–5) were fitted with fixed factors for both a linear and quadratic effect of relative neighbour group size. These models controlled for focal group size as a fixed factor, as well as being fit with random intercepts for focal group and neighbour group identity and random slopes for the effects of focal group size and relative neighbour group size within focal group identity. Random terms showing zero variance were kept in models, and the s.d. for these terms is noted as 0.000 in Supplementary Tables. To assess the effects of location within the home range on behaviour, we assigned all recorded behavioural observations a location within the focal group’s home range using the same GPS method as above. All observations within the 50% UD were assigned as occurring in the ‘core’ area, and all observations outside the 75% UD were assigned as occurring in the ‘edge’ area (Fig. 3a). Observations that fell in the buffer zone between core and edge (that is, 50–75% UD) were not analysed. Models investigating changes in behaviour relative to location within the home range (Supplementary Tables 6–8), not just restricted to neighbour zones, were fitted with fixed terms for absolute group size and location within the home range, a random intercept for group identity as well as random slopes for group size and location within-group identity. While groups consistently had unhabituated neighbours (for example, outer edges in Fig. 1a), our approaches are not impacted by these groups: for relative group-size analyses, we used only data from zones with known neighbours; the location-based analyses are egocentric and are therefore agnostic to whether a potential neighbour is in the habituated study population or not.

Before statistical modelling, we removed outliers (data points more than three s.d. from the mean) to enhance model fit and prevent models from violating assumptions. We scaled all absolute group-size, bout duration and overlap-size predictors to a mean of 0 and an s.d. of 1 to aid model fitting. We assessed significance of terms using likelihood ratio tests between the final model with and without the term of interest. Nonsignificant quadratic terms were removed to allow interpretation of the linear term—the model with significant quadratic term/without nonsignificant quadratic term was considered the final model, with all other a priori modelled predictors retained. Effect sizes are taken from the model summaries and transformed to the original data scale where appropriate.

All presented models were run using functions from the ‘lme4’^68^ (version 1.1–38) and ‘glmmTMB’^69^ (version 1.1.14) packages. We checked all model assumptions using diagnostic tools from the ‘DHARMa’ package^70^ (version 0.0.7), with variance inflation below 3 for all terms in all models, as checked using the vif() function from the ‘car’ package (version 3.1–5). We checked model residuals for temporal autocorrelation to ensure behaviour was not significantly correlated across days. In addition, we found little evidence that response metrics were correlated within group and day (Supplementary Fig. 3). The likelihood that a sentinel vocalized is weakly but significantly correlated with both the likelihood of a sentinel being present (*r* = 0.22, *P* < 0.001) and the duration of sentinel bouts (*r* = 0.17, *P* < 0.001). Models for vocalization include the log of bout duration as a covariate, as these variables are both gathered at the single bout level, determined by the log-linear relationship between bout durations and vocalization likelihood (Supplementary Fig. 4), whereas likelihood of a sentinel being present is from the independent scan-sample dataset. By nature, movement speed and area covered are also significantly correlated (*r* = 0.25, *P* < 0.001) but have the potential to highlight nuanced differences in movement behaviour and are therefore interpreted together^38^.

To assess the factors that influenced the extent to which groups overlap in their space use, we calculated 95% UDs for 3-month blocks (January–March, April–June, July–September and October–December) for all groups. We then calculated the size of areas that overlapped between neighbouring pairs, proportional to the full 95% UD of both groups. To control for potential impacts of environmental productivity on overlap size^38^, we took the 3-month mean normalized difference vegetation index value for the study area, taken from the MODIS product MOD13Q1^71^: 16-day, 250 m × 250 m resolution (as in ref. ^38^), using functions from the ‘MODISTools’ R package^72^ (version 1.1.6). Similarly, to control for population density, which could influence space use and overlap size^73^, we summed the size of all measured group 95% UDs and divided by the number of individuals over 1 year old to achieve a metric of adult mongooses per ha. For this analysis, absolute group size was calculated as the mean linear interpolation of the number of adult individuals across the 3-month block (mean of 7.9, s.d. of 2.0, range of 4.4–13.4, *N* = 274 group blocks). As each overlap can generate two proportional areas (overlap/group 1 versus overlap/group 2), we ran two near-identical models, from the perspective of the larger and smaller groups in each pair, respectively, to avoid pseudo-replication inflating our sample size. Both were modelled identically, with fixed factors included for normalized difference vegetation index, population density, time of year, mean group size (mean of the two neighbours in the pair) and the relative group size of the two neighbours. Both models yielded qualitatively identical results (Supplementary Table 1); we present those from the perspective of the larger group in the main text.

When assessing the likelihood of entering, and proportion of time spent within, neighbour zones, data were summarized at the level of the month. For each group, we took the proportion of days in which any fix was located within a neighbour zone with each neighbour, with a minimum of 5 days observed in a given month. We also analysed the proportion of all fixes observed in the neighbour zone with each specific neighbour, again with a minimum of five observation days per month. These were analysed using generalized linear mixed models (GLMMs) with gamma error and a log link function (Supplementary Table 2). All models contained an additional fixed factor for the proportional size of the overlap between the home ranges of the focal group with a given neighbour, relative to the home range size of the focal, to control for the effect of differences in mutual space use between groups.

We calculated movement speed for each fix (as distance/time since last fix) that was >50 and <70 s since its predecessor. The mean of each speed was then taken to generate the movement-speed metric. We determined unique area covered per unit time as the number of unique 20-m width hexagonal cells the track intersected divided by the time taken (the full method can be found in ref. ^38^. Both movement speed and unique area covered per unit time were modelled as linear mixed models (LMMs) with log-transformed response data (Supplementary Tables 3 and 6). Models investigating home range location contained observation day as an additional random intercept to capture the non-independence of track segments from within the same day (Supplementary Table 6); for this analysis, we only included days that had track segments in both core and edge areas.

Models investigating sentinel presence in a scan sample and whether a sentinel vocalized during a given bout (each with responses of yes or no; Supplementary Tables 4a,c and 7a,c) were analysed as GLMMs with a binomial error structure and logit link function. Models investigating the duration of sentinel bouts (Supplementary Tables 4b and 7b) were run as LMMs with the log-transformed bout duration fitted as the response term. Models investigating vocalization had bout duration (logged) fitted as an additional fixed term. All sentinel models contained an additional random intercept for the specific observation session to account for the non-independence of multiple data points taken in a single session.

We took all days in which a group was recorded entering a neighbour zone and noted whether they slept at a burrow in the neighbour zone that evening. This was modelled as a binomial GLMM with a logit link function (Supplementary Table 5a), with an additional fixed factor for the proportional size of the overlap between the focal group and given neighbour. Models investigating return time to the evening sleeping burrow (Supplementary Tables 5b and 8) were run as LMMs with log-transformed response data with a small constant added to all values before transformation to prevent negative values, thus enabling the transformation.

To help visualize our main effects, we added discretized raw data to our plots of model-estimated effects and CIs (Fig. 2). For models investigating relative group-size effects, where there is also a main effect of group size with *P* < 0.1 (Fig. 2b), we adjusted these plotted values by the estimated group-size effect, given the inherent relationship between absolute and relative group size (see Supplementary Fig. 1 for further details). Model output tables in Supplementary Information represent those values generated directly from running R code, whereas effect sizes and figures represent the back-transformation of these values, including accounting for any link functions in models and scaling of variables.

### Reporting summary

Further information on research design is available in the Nature Portfolio Reporting Summary linked to this article.

## Supplementary information

Supplementary Information Supplementary Figs. 1–4, Tables 1–10 and Analysis: location-based behaviour.

Reporting Summary

## Data Availability

All data required to reproduce analyses and figures in this article are available via figshare at 10.6084/m9.figshare.28574711 (ref. ^74^).

## Code availability

All code required to reproduce analyses and figures in this article are available via figshare at 10.6084/m9.figshare.28574711 (ref. ^74^).

## References

[1] Braga Goncalves, I., Morris-Drake, A., Kennedy, P. & Radford, A. N. Fitness consequences of outgroup conflict. *eLife* **11**, e74550 (2022). 35833830 10.7554/eLife.74550 PMC9282852

[2] Morris-Drake, A., Kennedy, P., Braga Goncalves, I. & Radford, A. N. Variation between species, populations, groups and individuals in the fitness consequences of out-group conflict. *Philos. Trans. R. Soc. B* **377**, 20210148 (2022). 10.1098/rstb.2021.0148 PMC8977661 35369741

[3] Lemoine, S. R. T., Samuni, L., Crockford, C. & Wittig, R. M. Parochial cooperation in wild chimpanzees: a model to explain the evolution of parochial altruism. *Philos. Trans. R. Soc. B* **377**, 20210149 (2022). 10.1098/rstb.2021.0149 PMC8977654 35369746

[4] Bowles, S. Did warfare among ancestral hunter-gatherers affect the evolution of human social behaviors?. *Science* **324**, 1293–1298 (2009). 19498163 10.1126/science.1168112

[5] West, S. A. et al. Cooperation and the scale of competition in humans. *Curr. Biol.* **16**, 1103–1106 (2006). 16753564 10.1016/j.cub.2006.03.069

[6] Johnstone, R. A., Cant, M. A., Cram, D. & Thompson, F. J. Exploitative leaders incite intergroup warfare in a social mammal. *Proc. Natl Acad. Sci. USA* **117**, 29759–29766 (2020). 33168743 10.1073/pnas.2003745117 PMC7703641

[7] Thompson, F. J., Marshall, H. H., Vitikainen, E. I. K. & Cant, M. A. Causes and consequences of intergroup conflict in cooperative banded mongooses. *Anim. Behav.* **126**, 31–40 (2017).

[8] Dyble, M., Houslay, T. M., Manser, M. B. & Clutton-Brock, T. Intergroup aggression in meerkats. *Proc. R. Soc. B* **286**, 20191993 (2019). 31847765 10.1098/rspb.2019.1993 PMC6939929

[9] Lemoine, S. et al. Between-group competition impacts reproductive success in wild chimpanzees. *Curr. Biol.* **30**, 312–318.e3 (2020). 31902731 10.1016/j.cub.2019.11.039 PMC6971690

[10] Schoof, V. A. M. & Jack, K. M. The association of intergroup encounters, dominance status, and fecal androgen and glucocorticoid profiles in wild male white-faced capuchins (*Cebus capucinus*). *Am. J. Primatol.* **75**, 107–115 (2013). 23090872 10.1002/ajp.22089 PMC3527667

[11] Crofoot, M. C. The cost of defeat: capuchin groups travel further, faster and later after losing conflicts with neighbors. *Am. J. Phys. Anthropol.* **152**, 79–85 (2013). 23900797 10.1002/ajpa.22330

[12] Schneider-Crease, I. A. et al. Beyond infant death: the hidden costs of male immigration in geladas. *Anim. Behav.* **159**, 89–95 (2020).

[13] Laundré, J. W., Hernández, L. & Altendorf, K. B. Wolves, elk, and bison: reestablishing the ‘landscape of fear’ in Yellowstone National Park, *U.S.A*. *Can. J. Zool.* **79**, 1401–1409 (2001).

[14] Wrangham, R., Lundy, R., Crofoot, M. & Gilby, I. Use of overlap zones among group-living primates: a test of the risk hypothesis. *Behaviour* **144**, 1599–1619 (2007).

[15] Radford, A. N. Preparing for battle? Potential intergroup conflict promotes current intragroup affiliation. *Biol. Lett.* **7**, 26–29 (2011). 20610419 10.1098/rsbl.2010.0507 PMC3030875

[16] Lemoine, S. R. T., Samuni, L., Crockford, C. & Wittig, R. M. Chimpanzees make tactical use of high elevation in territorial contexts. *PLoS Biol.* **21**, e3002350 (2023). 37917608 10.1371/journal.pbio.3002350 PMC10621857

[17] Gat, A. The pattern of fighting in simple, small-scale, prestate societies. *J. Anthropol. Res.* **55**, 563–583 (1999).

[18] Sample, S. G. Anticipating war? War preparations and the steps-to-war thesis. *Br. J. Polit. Sci.* **48**, 489–511 (2018).

[19] Manson, J. H. & Wrangham, R. W. Intergroup aggression in chimpanzees and humans. *Curr. Anthropol.* **32**, 369–377 (1991).

[20] Benadi, G., Fichtel, C. & Kappeler, P. Intergroup relations and home range use in Verreaux’s sifaka (*Propithecus verreauxi*). *Am. J. Primatol.* **70**, 956–965 (2008). 18613007 10.1002/ajp.20588

[21] Tórrez-Herrera, L. L., Davis, G. H. & Crofoot, M. C. Do monkeys avoid areas of home range overlap because they are dangerous? A test of the risk hypothesis in white-faced capuchin monkeys (*Cebus capucinus*). *Int. J. Primatol.* **41**, 246–264 (2020).

[22] Wilson, M., Hauser, M. & Wrangham, R. Chimpanzees (*Pan troglodytes*) modify grouping and vocal behaviour in response to location-specific risk. *Behaviour* **144**, 1621–1653 (2007).

[23] Green, P. A., Thompson, F. J. & Cant, M. A. Fighting force and experience combine to determine contest success in a warlike mammal. *Proc. Natl Acad. Sci. USA* **119**, e2119176119 (2022). 35700363 10.1073/pnas.2119176119 PMC9231503

[24] Majolo, B., deBortoli Vizioli, A., Martínez-Íñigo, L. & Lehmann, J. Effect of group size and individual characteristics on intergroup encounters in primates. *Int. J. Primatol.* **41**, 325–341 (2020).

[25] Radford, A. N. Territorial vocal rallying in the green woodhoopoe: influence of rival group size and composition. *Anim. Behav.* **66**, 1035–1044 (2003).

[26] Roth, A. M. & Cords, M. Effects of group size and contest location on the outcome and intensity of intergroup contests in wild blue monkeys. *Anim. Behav.* **113**, 49–58 (2016).

[27] Briffa, M. What determines the duration of war? Insights from assessment strategies in animal contests. *PLoS ONE* **9**, e108491 (2014). 25247403 10.1371/journal.pone.0108491 PMC4172633

[28] Morrison, R. E. et al. Inter-group relationships influence territorial defence in mountain gorillas. *J. Anim. Ecol.* **89**, 2852–2862 (2020). 33111342 10.1111/1365-2656.13355

[29] Yi, Y., Fichtel, C., Ham, S., Jang, H. & Choe, J. C. Fighting for what it’s worth: participation and outcome of inter-group encounters in a pair-living primate, the Javan gibbon (*Hylobates moloch*). *Behav. Ecol. Sociobiol.* **74**, 96 (2020).

[30] Jordan, N. R., Mwanguhya, F., Kyabulima, S., Rüedi, P. & Cant, M. A. Scent marking within and between groups of wild banded mongooses. *J. Zool.* **280**, 72–83 (2010).

[31] Mitani, J. C., Watts, D. P. & Amsler, S. J. Lethal intergroup aggression leads to territorial expansion in wild chimpanzees. *Curr. Biol.* **20**, R507–R508 (2010). 20620900 10.1016/j.cub.2010.04.021

[32] Arbon, J. J. et al. Life-history and genetic relationships in cooperatively breeding dwarf mongoose groups. *R. Soc. Open Sci.* **11**, 241125 (2024). 39359473 10.1098/rsos.241125 PMC11444783

[33] Christensen, C., Kern, J. M., Bennitt, E. & Radford, A. N. Rival group scent induces changes in dwarf mongoose immediate behavior and subsequent movement. *Behav. Ecol.* **27**, 1627–1634 (2016).

[34] Morris-Drake, A., Cobb, B., Kern, J. M. & Radford, A. N. A positive effect of cumulative intergroup threat on reproductive success. *Proc. R. Soc. B* **290**, 20231853 (2023). 37964527 10.1098/rspb.2023.1853 PMC10646463

[35] Kern, J. M. & Radford, A. N. Sentinel dwarf mongooses, *Helogale parvula*, exhibit flexible decision making in relation to predation risk. *Anim. Behav.* **98**, 185–192 (2014).

[36] Morris-Drake, A., Christensen, C., Kern, J. M. & Radford, A. N. Experimental field evidence that out-group threats influence within-group behavior. *Behav. Ecol.* **30**, 1425–1435 (2019). 31579132 10.1093/beheco/arz095 PMC6765380

[37] Kern, J. M. & Radford, A. N. Call of duty? Variation in use of the watchman’s song by sentinel dwarf mongooses, *Helogale parvula*. *Anim. Behav.* **85**, 967–975 (2013).

[38] Arbon, J. J., Morris-Drake, A., Kern, J. M., Giuggioli, L. & Radford, A. N. Social and seasonal variation in dwarf mongoose home-range size, daily movements, and burrow use. *Behav. Ecol.* **35**, arae082 (2024). 39473884 10.1093/beheco/arae082 PMC11520750

[39] Kern, J. M., Sumner, S. & Radford, A. N. Sentinel dominance status influences forager use of social information. *Behav. Ecol.* **27**, 1053–1060 (2016).

[40] Kern, J. M. & Radford, A. N. Experimental evidence for delayed contingent cooperation among wild dwarf mongooses. *Proc. Natl Acad. Sci. USA* **115**, 6255–6260 (2018). 29844179 10.1073/pnas.1801000115 PMC6004489

[41] Morris-Drake, A., Kern, J. M. & Radford, A. N. Experimental evidence for delayed post-conflict management behaviour in wild dwarf mongooses. *eLife* **10**, e69196 (2021). 34725038 10.7554/eLife.69196 PMC8562999

[42] Arbon, J. J., Kern, J. M., Morris-Drake, A. & Radford, A. N. Context-dependent contributions to sentinel behaviour: audience, satiation and danger effects. *Anim. Behav.* **165**, 143–152 (2020).

[43] Rasa, O. A. E. Marking behaviour and its social significance in the African dwarf mongoose, *Helogale undulata rufula*. *Z. Tierpsychol.* **32**, 293–318 (1973). 4781185

[44] Sharpe, L. L., Jooste, M. M. & Cherry, M. I. Handstand scent marking in the dwarf mongoose (*Helogale parvula*). *Ethology* **118**, 575–583 (2012).

[45] Strandburg-Peshkin, A., Clutton-Brock, T. & Manser, M. B. Burrow usage patterns and decision-making in meerkat groups. *Behav. Ecol*. 10.1093/beheco/arz190 (2019).

[46] Reyna-Hurtado, R. et al. Primates adjust movement strategies due to changing food availability. *Behav. Ecol.* **29**, 368–376 (2018).

[47] Zanette, L. Y. & Clinchy, M. Ecology and neurobiology of fear in free-living wildlife. *Annu. Rev. Ecol. Evol. Syst.* **51**, 297–318 (2020).

[48] Creel, S., Christianson, D., Liley, S. & Winnie, J. A. Predation risk affects reproductive physiology and demography of elk. *Science* **315**, 960 (2007). 17303746 10.1126/science.1135918

[49] Morris-Drake, A., Linden, J. F., Kern, J. M. & Radford, A. N. Extended and cumulative effects of experimentally induced intergroup conflict in a cooperatively breeding mammal. *Proc. R. Soc. B* **288**, 20211743 (2021). 34875195 10.1098/rspb.2021.1743 PMC8651417

[50] Creel, S. in *Carnivores, Pangolins Equids and Rhinoceroses* (eds Kingdon, J. & Hoffman, M.) 368–373 (Bloomsbury, 2013).

[51] Aureli, F. et al. Fission-fusion dynamics. *Curr. Anthropol.* **49**, 627–654 (2008).

[52] Ashton, B. J., Kennedy, P. & Radford, A. N. Interactions with conspecific outsiders as drivers of cognitive evolution. *Nat. Commun.* **11**, 4937 (2020). 33024110 10.1038/s41467-020-18780-3 PMC7538913

[53] Ray, S. et al. Hippocampal coding of identity, sex, hierarchy, and affiliation in a social group of wild fruit bats. *Science* **387**, eadk9385 (2025). 39883756 10.1126/science.adk9385

[54] Koch, F., Signer, J., Kappeler, P. M. & Fichtel, C. Intergroup encounters in Verreaux’s sifakas (*Propithecus verreauxi*): who fights and why?. *Behav. Ecol. Sociobiol.* **70**, 797–808 (2016). 27194822 10.1007/s00265-016-2105-3 PMC4841837

[55] Jacobson, O. T., Crofoot, M. C., Finerty, G. E., Perry, S. E. & Barrett, B. J. Environmental fluctuations alter the competitive trade-offs of group size in a social primate. *Nat. Ecol. Evol.* **10**, 919–931 (2026). 42091685 10.1038/s41559-026-03048-8 PMC13167455

[56] Watts, H. E. & Holekamp, K. E. Hyena societies. *Curr. Biol.* **17**, R657–R660 (2007). 17714659 10.1016/j.cub.2007.06.002

[57] Watts, D. P. & Mitani, J. C. Boundary patrols and intergroup encounters in wild chimpanzees. *Behaviour* **138**, 299–327 (2001).

[58] Herdtle, A., Duncan, C., Manser, M. B. & Clutton-Brock, T. Patterns and drivers of female extra-pair mating in wild Kalahari meerkats. *Behav. Ecol.* **36**, araf016 (2025). 40171036 10.1093/beheco/araf016 PMC11959362

[59] Rasa, O. A. E. in *Advances in the Study of Behavior*, Vol. 17 121–163 (Elsevier, 1987).

[60] Rasa, O. A. E. Coordinated vigilance in dwarf mongoose family groups: the ‘watchman’s song’ hypothesis and the costs of guarding. *Ethology* **71**, 340–344 (1986).

[61] Hoffmann, M., Roberts, R. L. & Kern, J. Tree climbing and denning by common dwarf mongoose. *Small Carniv. Conserv.* **50**, 66–67 (2014).

[62] ASAB Guidelines for the treatment of animals in behavioural research and teaching. *Anim. Behav.* **159**, i–xi (2020). 10.1006/anbe.1999.1349 10640387

[63] R Core Team *R: A Language and Environment for Statistical Computing* (R Foundation for Statistical Computing, 2025).

[64] Langley, L. P., Lang, S. D. J., Ozsanlav-Harris, L. & Trevail, A. M. ExMove: an open-source toolkit for processing and exploring animal-tracking data in R. *J. Anim. Ecol*. 10.1111/1365-2656.14111 (2024). 10.1111/1365-2656.14111 38860632

[65] Calenge, C. & Fortmann-Roe, S. adehabitatHR: home range estimation (2023).

[66] Calabrese, J. M., Fleming, C. H. & Gurarie, E. ctmm: an R package for analyzing animal relocation data as a continuous-time stochastic process. *Methods Ecol. Evol.* **7**, 1124–1132 (2016).

[67] Pebesma, E. Simple features for R: standardized support for spatial vector data. *R J.* **10**, 439–446 (2018).

[68] Bates, D., Mächler, M., Bolker, B. & Walker, S. Fitting linear mixed-effects models using lme4. *J. Stat. Softw.* **67**, 1–48 (2015).

[69] Brooks, M. E. et al. glmmTMB balances speed and flexibility among packages for zero-inflated generalized linear mixed modeling. *R J.* **9**, 378–400 (2017).

[70] Hartig, F. DHARMa: residual diagnostics for hierarchical (multi-level/mixed) regression models (2022).

[71] Didan, K. MODIS/Terra Vegetation Indices 16-day L3 global 250m SIN Grid V061 (dataset). *NASA EOSDIS Land Processes Distributed Active Archive Center*10.5067/MODIS/MOD13Q1.061 (2021).

[72] Hufkens, K. The MODISTools package: an interface to the MODIS Land Products Subsets Web Services (2023).

[73] Vander Wal, E., Laforge, M. P. & McLoughlin, P. D. Density dependence in social behaviour: home range overlap and density interacts to affect conspecific encounter rates in a gregarious ungulate. *Behav. Ecol. Sociobiol.* **68**, 383–390 (2014).

[74] Arbon, J. J., Morris-Drake, A., Kern, J. M. & Radford, A. N. Data and code for: Dwarf mongooses pre-emptively alter their behaviour relative to the threat posed by different rival groups. *figshare*10.6084/m9.figshare.28574711 (2026). 10.1038/s41559-026-03104-3 PMC13441901 42303729

