# Supplementary material for: Dwarf mongooses pre-emptively alter their behaviour relative to the threat posed by different rival groups
Source: Nat Ecol Evol. 2026 Jun 16;10(8):1521–30. doi: 10.1038/s41559-026-03104-3 (PMC13441901; doi:10.1038/s41559-026-03104-3)
Supplement: Supplementary file 1 [file 41559_2026_3104_MOESM1_ESM.pdf]

nature ecology & evolution

Supplementary information

https://doi.org/10.1038/s41559-026-03104-3

Dwarf mongooses pre-emptively alter their behaviour relative to the threat posed by different rival groups

In the format provided by the authors and unedited

Table of Contents:

2–5: Figs S1 to S4
6–12: Tables S1 to S10
13–16: Supplementary Analysis: Location-based Behaviour

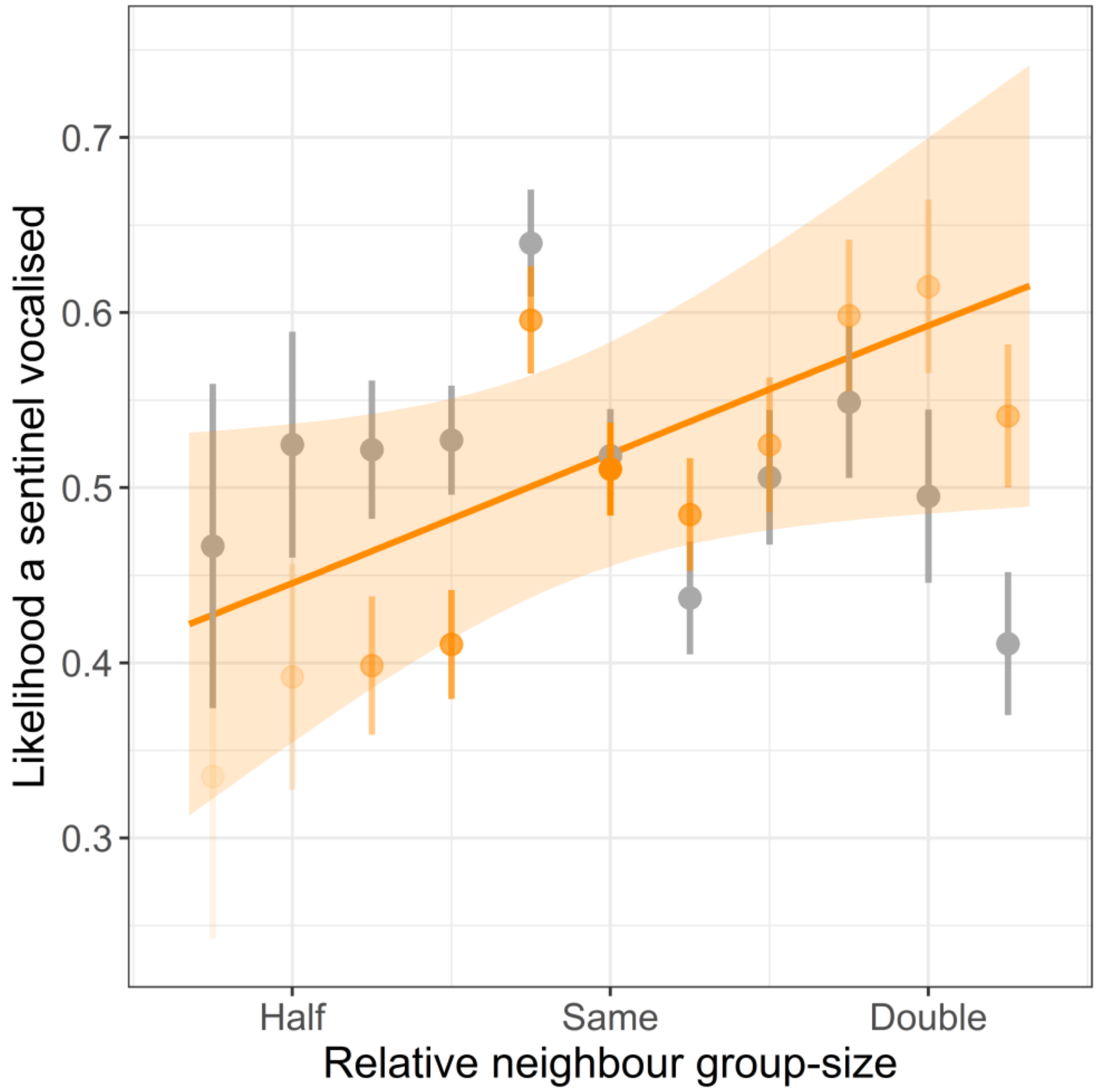

Supplementary Figure 1. Accounting for marginal effects of absolute group-size helps reveal relative neighbour group-size effect. The likelihood of a sentinel vocalising present relative to neighbour group-size. Plot is the same as Figure 2C in the main text, with the addition of the raw data (grey points). Orange points account for the indirect relationship between relative neighbour group-size and the likelihood of a sentinel vocalising that is due to differences in group size affecting vocalisation likelihood. As sentinels in larger groups (in an absolute sense) are more likely to vocalise (Supplementary Table 3C), and these larger groups are more likely by nature to have smaller relative neighbours, plotting the raw data (grey points) does not account for this indirect relationship and generates a mismatch between the model predictions and plotted data. Orange (corrected) points are calculated by multiplying the raw values by the model derived absolute group-size effect proportional to the difference between the average group-size for each relative neighbour group-size bin and the overall mean absolute group size. N = 1936 bouts.

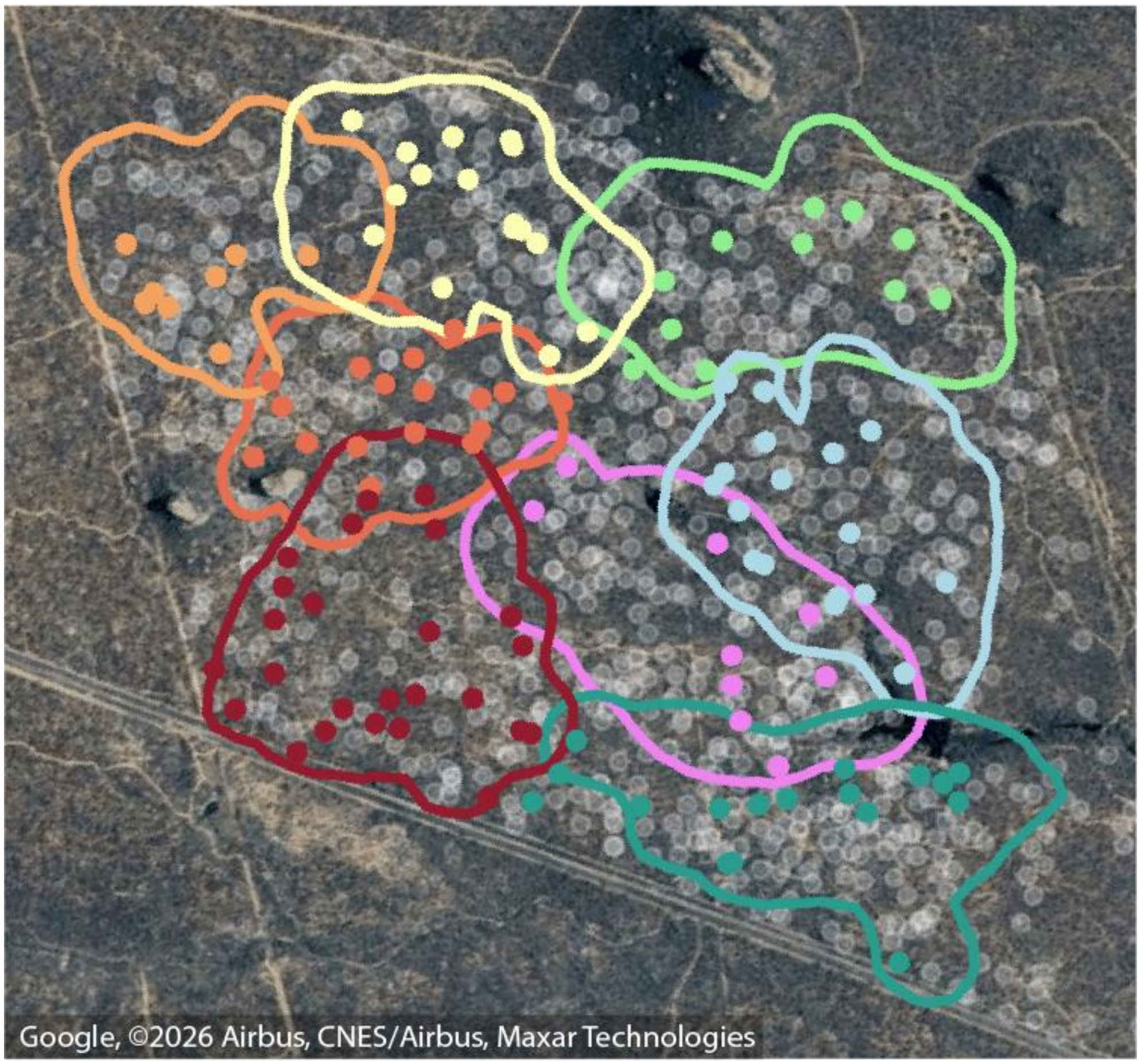

Supplementary Figure 2. Burrows are numerous and distributed across the landscape. Map of the homeranges of the eight study groups for same period as Figure 1A (2018 non-breeding season), with each group denoted by a different colour. Coloured points are all burrows that were observed being used by each group during this period, whilst grey points are all burrows that have been observed to be used over the course of the history of the Dwarf Mongoose Research Project. N = 8 groups.

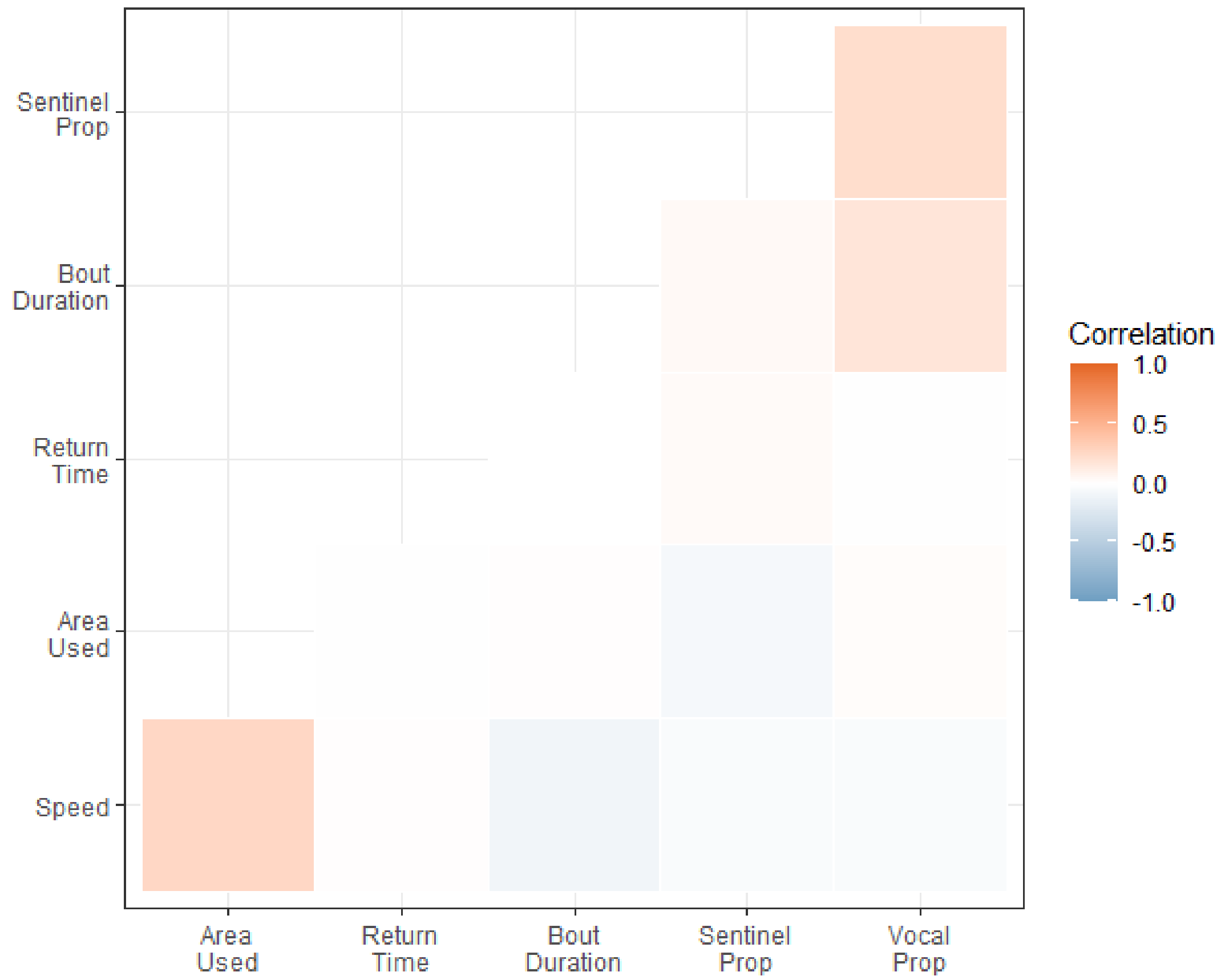

Supplementary Figure 3. Response metrics were not strongly correlated across behaviours. Correlation coefficients between response metrics from days in which a group was within a neighbour zone and all metrics were available from the same group. Intersecting areas represent the correlation between the two responses. As highlighted in pink, there were weak correlations between sentinel metrics (proportion of scan-samples with a sentinel, duration of sentinel bouts, proportion of bouts that were announced with vocalisations), as well as between the two movement metrics (movement speed and unique area used per unit time), but not between any other pair of metrics. N = 653 group-days.

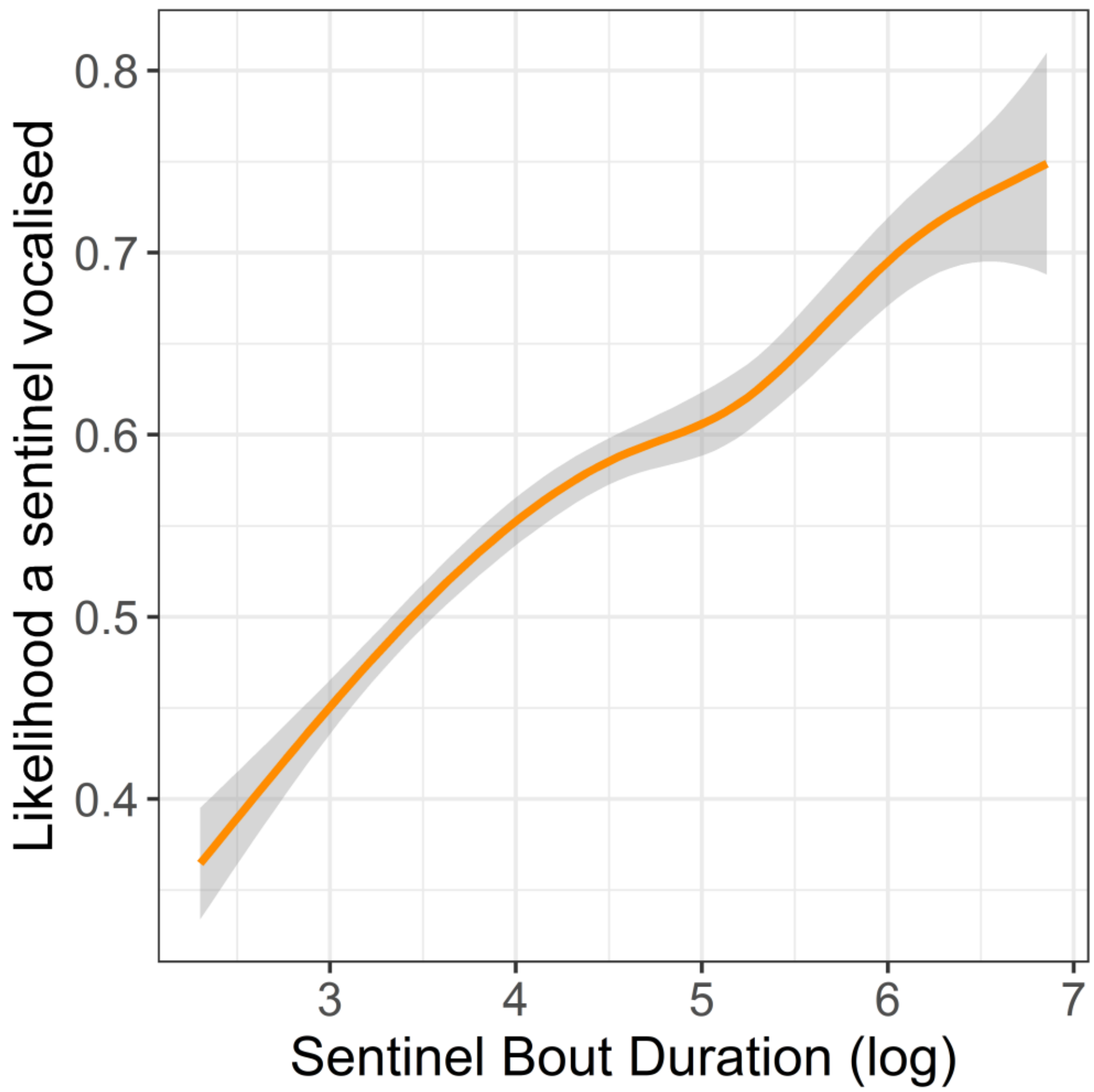

Supplementary Figure 4. The relationship between the duration of sentinel bouts and the likelihood that a sentinel vocalised. Sentinels are more likely to vocalise when they conduct longer bouts, and this relationship is best described as log-linear. N=12490 sentinel bouts.

Supplementary Table 1. Output of a beta regression investigating the factors that influence the proportion of homerange overlap by neighbouring groups. Outputs from the perspective of the (A) larger group (as reported in main text) and (B) smaller group are qualitatively identical. Confidence intervals (CI) are estimated as 95% from 1000 bootstraps, significant fixed factors in bold. N=135 overlaps, each over a 3-month block.

Factor	Estimate	95% CI	χ^2	DoF	P
(A) Larger Group					
Intercept	-3.116	-4.051, -2.181			
Mean Group-Size	0.095	0.020, 0.170	6.033	1	**0.014**
Relative Neighbour Group-Size	-0.118	-1.253, 1.018	0.041	1	0.839
Greenness (NDVI)	0.157	-0.152, 0.467	0.996	1	0.318
Population Density	0.006	-0.151, 0.163	0.005	1	0.942
Time of Year			3.166	3	0.367
Time of $Year_{Apr–Jun}$	0.040	-0.486, 0.566			
Time of $Year_{Jul–Sep}$	0.448	-0.403, 1.298			
Time of $Year_{Oct–Dec}$	-0.008	-0.629, 0.612			
(B) Smaller Group					
Intercept	-2.983	-3.898, -2.067			
Mean Group-Size	0.077	0.005, 0.149	4.332	1	**0.037**
Relative Neighbour Group-Size	-0.770	-1.907, 0.367	1.803	1	0.179
Greenness (NDVI)	0.100	-0.205, 0.405	0.414	1	0.520
Population Density	0.013	-0.140, 0.167	0.029	1	0.864
Time of Year			1.775	3	0.620
Time of $Year_{Apr–Jun}$	-0.019	-0.546, 0.508			
Time of $Year_{Jul–Sep}$	0.324	-0.526, 1.174			
Time of $Year_{Oct–Dec}$	0.026	-0.592, 0.643			

Supplementary Table 2. Output of models investigating space use relative to neighbour zones measured as (A) the proportion of days in which a group entered a neighbour zone and (B) the proportion of their time (proportion of GPS fixes) that they spent within a neighbour zone. Analysed as gamma Generalised Linear Mixed Models with a log link function. Confidence intervals (CI) are estimated as 95% from 1000 bootstraps, random term standard deviation italicised, significant fixed factors in bold. N=511 observations from 340 unique group-months of 11 focal groups relative to 12 neighbours.

Factor	Estimate	95% CI	χ^2	DoF	P
(A) Number of Days					
Intercept	-1.283	-1.418, -1.172			
Absolute Group-Size	0.085	0.019, 0.158	3.148	1	0.076
Relative Neighbour Group-Size	0.055	-0.063, 0.167	1.194	1	0.274
Relative Neighbour Group-Size^2			0.733	1	0.392
Overlap Size	0.312	0.258, 0.368	120.112	1	**<0.001**
Group ID $_{Intercept}$	*0.048*	*2.194e^{-5}, 0.271*			
Group ID $_{Absolute\ Group\text{-}Size}$	*5.652e^{-6}*	*3.510e^{-6}, 0.184*			
Group ID $_{Relative\ Neighbour\ Group\text{-}Size}$	*0.067*	*0.036, 0.293*			
Neighbour ID $_{Intercept}$	*0.071*	*0.082, 0.321*			
(B) Number of Fixes					
Intercept	-2.279	-2.391, -2.183			
Absolute Group-Size	0.000	-0.129, 0.117	0.000	1	0.995
Relative Neighbour Group-Size	-0.071	-0.196, 0.049	1.360	1	0.244
Relative Neighbour Group-Size^2	-0.104	-0.172, -0.044	8.722	1	**0.003**
Overlap Size	0.614	0.523, 0.706	128.303	1	**<0.001**
Group ID $_{Intercept}$	*2.133e^{-5}*	*2.712e^{-7}, 0.147*			
Group ID $_{Absolute\ Group\text{-}Size}$	*0.000*	*0.000, 0.133*			
Group ID $_{Relative\ Neighbour\ Group\text{-}Size}$	*1.849e^{-5}*	*5.313e^{-7}, 0.139*			
Neighbour ID $_{Intercept}$	*0.000*	*1.034e^{-6}, 1.130*			

Supplementary Table 3. Output of models investigating (A) movement speed and (B) unique area covered per unit time within neighbour zones depending on relative neighbour group-size. Analysed as Linear Mixed Models with log-transformed response data. Confidence intervals (CI) are estimated as 95% from 1000 bootstraps, random term standard deviation italicised, significant fixed factors in bold. N=1237 observations of 11 focal groups relative to 12 neighbours.

Factor	Estimate	95% CI	χ^2	DoF	P
(A) Movement Speed					
Intercept	-2.681	-2.729, -2.639			
Absolute Group-Size	-0.002	-0.041, 0.038	0.003	1	0.958
Relative Neighbour Group-Size	-0.074	-0.282, 0.128	0.523	1	0.470
Relative Neighbour Group-Size^2			1.429	1	0.232
Overlap Size	0.010	-0.019, 0.042	0.422	1	0.516
Group ID $_{Intercept}$	*0.000*	*0.000, 0.052*			
Group ID $_{Absolute\ Group\text{-}Size}$	*0.015*	*0.000, 0.065*			
Group ID $_{Relative\ Neighbour\ Group\text{-}Size}$	*0.153*	*0.000, 0.322*			
Neighbour ID $_{Intercept}$	*0.050*	*0.000, 0.097*			
(B) Area Covered per Unit Time					
Intercept	-1.651	-1.741, -1.566			
Absolute Group-Size	-0.037	-0.138, 0.071	0.417	1	0.519
Relative Neighbour Group-Size	-0.052	-0.557, 0.446	0.041	1	0.839
Relative Neighbour Group-Size^2			0.127	1	0.723
Overlap Size	-0.025	-0.078, 0.029	0.818	1	0.366
Group ID $_{Intercept}$	*0.064*	*0.000, 0.150*			
Group ID $_{Absolute\ Group\text{-}Size}$	*0.121*	*0.000, 0.209*			
Group ID $_{Relative\ Neighbour\ Group\text{-}Size}$	*0.510*	*0.000, 0.906*			
Neighbour ID $_{Intercept}$	*0.066*	*0.000, 0.136*			

Supplementary Table 4. Output of models investigating characteristics of sentinel behaviour in neighbour zones depending on relative neighbour group-size: (A) likelihood that a sentinel was present, (B) sentinel bout duration and (C) likelihood that sentinel vocalised to announce presence. (A,C) Analysed as Generalised Linear Mixed Models with a binomial error structure and logit link function, (B) analysed as Linear Mixed Models with log-transformed response data. Confidence intervals (CI) are estimated as 95% from 1000 bootstraps, random term standard deviation italicised, significant fixed factors in bold. (A) N=3328 scan-samples across 1083 observation sessions with 11 groups relative to 12 neighbours, (B) N=2057 sentinel bouts across 677 observation sessions with 11 groups relative to 11 neighbours, (C) N=1936 sentinel bouts across 655 observation sessions with 11 groups relative to 11 neighbours.

Factor	Estimate	95% CI	χ^2	DoF	P
(A) Sentinel Presence					
Intercept	-0.214	-0.575, 0.133			
Absolute Group-Size	0.311	-0.063, 0.653	2.166	1	0.141
Relative Neighbour Group-Size	0.124	-0.044, 0.288	1.878	1	0.171
Relative Neighbour Group-Size^2			0.612	1	0.434
Observation Session$_{Intercept}$	*0.753*	*0.508, 0.786*			
Group ID $_{Intercept}$	*0.452*	*4.199e^{-4}, 0.689*			
Group ID $_{Absolute\ Group\text{-}Size}$	*0.508*	*2.567e^{-4}, 0.741*			
Group ID $_{Relative\ Neighbour\ Group\text{-}Size}$	*0.145*	*1.087e^{-6}, 0.315*			
Neighbour ID $_{Intercept}$	*0.234*	*3.752e^{-6}, 0.402*			
(B) Sentinel Bout Duration					
Intercept	4.251	4.134, 4.353			
Absolute Group-Size	-0.099	-0.248, 0.029	2.487	1	0.115
Relative Neighbour Group-Size	-0.198	-0.922, 0.454	0.425	1	0.514
Relative Neighbour Group-Size^2			0.142	1	0.706
Observation Session$_{Intercept}$	*0.401*	*0.330, 0.663*			
Group ID $_{Intercept}$	*0.091*	*0.000, 0.180*			
Group ID $_{Absolute\ Group\text{-}Size}$	*0.143*	*0.000, 0.258*			
Group ID $_{Relative\ Neighbour\ Group\text{-}Size}$	*0.799*	*6.780e^{-7}, 1.337*			
Neighbour ID $_{Intercept}$	*0.037*	*0.000, 0.147*			
(C) Sentinel Vocalisation					
Intercept	0.087	-0.241, 0.406			
Absolute Group-Size	0.462	0.233, 0.657	12.201	1	**<0.001**
Relative Neighbour Group-Size	0.255	0.022, 0.462	4.133	1	**0.042**
Relative Neighbour Group-Size^2			3.744	1	0.053
log(Bout Duration)	0.617	0.493, 0.737	103.457	1	**<0.001**
Observation Session$_{Intercept}$	*1.056*	*0.739, 1.104*			
Group ID $_{Intercept}$	*0.202*	*0.000, 0.414*			
Group ID $_{Absolute\ Group\text{-}Size}$	*0.000*	*0.000, 0.255*			
Group ID $_{Relative\ Neighbour\ Group\text{-}Size}$	*0.158*	*0.000, 0.323*			
Neighbour ID $_{Intercept}$	*0.316*	*0.000, 0.532*			

Supplementary Table 5. Output of models investigating behaviour relative to overnight sleeping burrows in neighbour zones depending on relative neighbour group size: (A) likelihood of sleeping in the neighbour zone on days in which the group entered the zone and (B) return time to the evening sleeping burrow. (A) Analysed using Generalised Linear Mixed Models with a binomial error structure and logit link function, (B) analysed as Linear Mixed Models with log-transformed response data; a constant was applied to all response values for (B) before transformation to remove negative values. Confidence intervals (CI) are estimated as 95% from 1000 bootstraps, random term standard deviation italicised, significant fixed factors in bold. (A) N=893 observations of 11 groups relative to 12 neighbours and sleeping in a known location that evening, (B) N=315 returns to evening burrows from 11 groups relative to 11 neighbours.

Factor	Estimate	95% CI	χ^2	DoF	P
(A) Sleeping in Neighbour Zone					
Intercept	-0.967	-1.463, -0.592			
Absolute Group-Size	0.159	-0.046, 0.378	2.114	1	0.146
Relative Neighbour Group-Size	0.133				
Relative Neighbour Group-Size^2	0.178	0.026, 0.342	4.846	1	**0.028**
Overlap Size	0.550	0.374, 0.751	36.016	1	**<0.001**
Group ID $_{Intercept}$	*0.486*	*0.000, 0.809*			
Group ID $_{Absolute\ Group\text{-}Size}$	*1.526e^{-7}*	*5.334e^{-8}, 2.518e^{-7}*			
Group ID $_{Relative\ Neighbour\ Group\text{-}Size}$	*0.446*	*0.000, 0.707*			
Neighbour ID $_{Intercept}$	*0.200*	*0.000, 0.506*			
(B) Return Time to Evening Burrow					
Intercept	3.887	3.801, 3.967			
Absolute Group-Size	-0.081	-0.208, 0.059	1.819	1	0.177
Relative Neighbour Group-Size	-0.395	-1.143, 0.361	1.161	1	0.281
Relative Neighbour Group-Size^2			2.739	1	0.098
Group ID $_{Intercept}$	*0.000*	*0.000, 0.143*			
Group ID $_{Absolute\ Group\text{-}Size}$	*0.112*	*0.000, 0.235*			
Group ID $_{Relative\ Neighbour\ Group\text{-}Size}$	*0.858*	*0.000, 1.494*			
Neighbour ID $_{Intercept}$	*0.035*	*0.000, 0.144*			

Supplementary Table 6. Output of models investigating (A) movement speed and (B) unique area covered per unit time relative to the location within the group's homerange, analysed as Linear Mixed Models with log-transformed response data. Confidence intervals (CI) are estimated as 95% from 1000 bootstraps, random term standard deviation italicised, significant fixed factors in bold. N=3304 track segments, N=1653 observation days of 12 groups.

Factor	Estimate	95% CI	χ^2	DoF	P
(A) Movement Speed					
Intercept	-2.631	-2.671, -2.585			
Absolute Group-Size	0.017	-0.008, 0.040	2.582	1	0.108
Homerange Location $_{Edge}$	0.162	0.112, 0.209	20.807	1	**<0.001**
Observation Day	*0.227*	*0.199, 0.239*			
Group ID $_{Intercept}$	*0.050*	*0.000, 0.082*			
Group ID $_{Absolute\ Group\ Size}$	*0.018*	*0.000,0.047*			
Group ID $_{Location\ -\ Core}$	*0.024*	*0.000, 0.081*			
Group ID $_{Location\ -\ Edge}$	*0.044*	*0.010, 0.092*			
(B) Area Covered per Unit Time					
Intercept	-1.654	-1.713, -1.583			
Absolute Group-Size	0.054	0.024, 0.087	7.697	1	**0.006**
Homerange Location $_{Edge}$	0.335	0.256, 0.408	25.128	1	**<0.001**
Observation Day	*0.221*	*0.169,0.259*			
Group ID $_{Intercept}$	*0.079*	*$1.151e^{-6}$, 0.119*			
Group ID $_{Absolute\ Group\ Size}$	*0.021*	*0.000, 0.566*			
Group ID $_{Location\ -\ Core}$	*0.051*	*0.009, 0.125*			
Group ID $_{Location\ -\ Edge}$	*0.079*	*0.014, 0.140*			

Supplementary Table 7. Output of models investigating characteristics of sentinel behaviour relative to the location within the group's homerange: (A) likelihood that a sentinel was present, (B) sentinel bout duration and (C) likelihood that sentinel vocalised to announce presence. (A,C) Analysed as Generalised Linear Mixed Models with a binomial error structure and logit link function, (B) analysed as Linear Mixed Models with log-transformed response data. Confidence intervals (CI) are estimated as 95% from 1000 bootstraps, random term standard deviation italicised, significant fixed factors in bold. (A) N=16660 scan-samples across 4181 observation sessions with 12 groups, (B) N=9950 sentinel bouts across 2855 observation sessions with 12 groups, (C) N=9493 sentinel bouts across 2756 observation sessions with 12 groups.

Factor	Estimate	95% CI	χ^2	DoF	P
(A) Sentinel Presence					
Intercept	0.035	-0.293, 0.364			
Absolute Group-Size	0.314	-0.012, 0.621	3.429	1	0.064
Homerange Location $_{Edge}$	-0.223	-0.331, -0.102	9.217	1	**0.002**
Observation Day	*0.825*	*0.689, 0.805*			
Group ID $_{Intercept}$	*0.002*	*5.411e^{-6},0.372*			
Group ID $_{Absolute\ Group\ Size}$	*0.501*	*0.226, 0.696*			
Group ID $_{Location\ -\ Core}$	*0.560*	*0.208, 0.780*			
Group ID $_{Location\ -\ Edge}$	*0.632*	*0.235, 0.890*			
(B) Sentinel Bout Duration					
Intercept	4.275	4.210, 4.351			
Absolute Group-Size	-0.092	-0.172, -0.007	4.161	1	**0.041**
Homerange Location $_{Edge}$	-0.146	-0.206, -0.091	11.659	1	**<0.001**
Observation Day	*0.445*	*0.416, 0.474*			
Group ID $_{Intercept}$	*1.976e^{-5}*	*0.000, 0.059*			
Group ID $_{Absolute\ Group\ Size}$	*0.117*	*0.032, 0.172*			
Group ID $_{Location\ -\ Core}$	*0.097*	*0.106, 0.569*			
Group ID $_{Location\ -\ Edge}$	*0.113*	*0.153, 0.724*			
(C) Sentinel Vocalisation					
Intercept	0.359	0.075, 0.598			
Absolute Group-Size	0.288	0.114, 0.444	6.930	1	**0.008**
Homerange Location $_{Edge}$	0.089	-0.053, 0.240	1.120	1	0.290
log(Bout Duration)	0.509	0.445, 0.563	347.310	1	**<0.001**
Observation Day	*1.091*	*0.897, 1.073*			
Group ID $_{Intercept}$	*0.003*	*2.953e^{-5}, 0.270*			
Group ID $_{Absolute\ Group\ Size}$	*0.216*	*2.776e^{-5}, 0.337*			
Group ID $_{Location\ -\ Core}$	*0.402*	*0.109, 0.569*			
Group ID $_{Location\ -\ Edge}$	*0.500*	*0.153, 0.724*			

Supplementary Table 8. Output of Linear Mixed Models with log-transformed response data on return time to the evening sleeping burrow relative to the location within the group's homerange. A constant was applied to all response values before transformation to remove negative values. Confidence intervals (CI) are estimated as 95% from 1000 bootstraps, random term standard deviation italicised, significant fixed factors in bold. N=2013 returns to evening burrows across 12 groups.

Factor	Estimate	95% CI	χ^2	DoF	P
Intercept	3.994	3.955, 4.038			
Absolute Group-Size	-0.045	-0.077, -0.013	5.385	1	**0.020**
Homerange $\text{Location}_{\text{Edge}}$	-0.123	-0.209, -0.032	5.872	1	**0.015**
Group ID $_{\textit{Intercept}}$	0.040	0.000, 0.065			
Group ID $_{\textit{Absolute Group Size}}$	0.030	0.000, 0.066			
Group ID $_{\textit{Location - Core}}$	0.021	0.000, 0.070			
Group ID $_{\textit{Location - Edge}}$	*0.120*	*0.028, 0.204*			

Supplementary Analysis: Location-based Behaviour. Sentinel and burrow observations were also analysed by assigning each observation to the minimum 5% utilisation distribution (UD) it fell in (e.g. observations within the 70% UD are also within the 75%, 80% etc. and therefore assigned to the 70%). All observations within the 50% UD were assigned to the same grouping, whilst all behaviour outside the 95% UD was assigned to the 100% UD. Results are qualitatively the same as analyses presented in the main text comparing core vs edge areas (Figure 3).

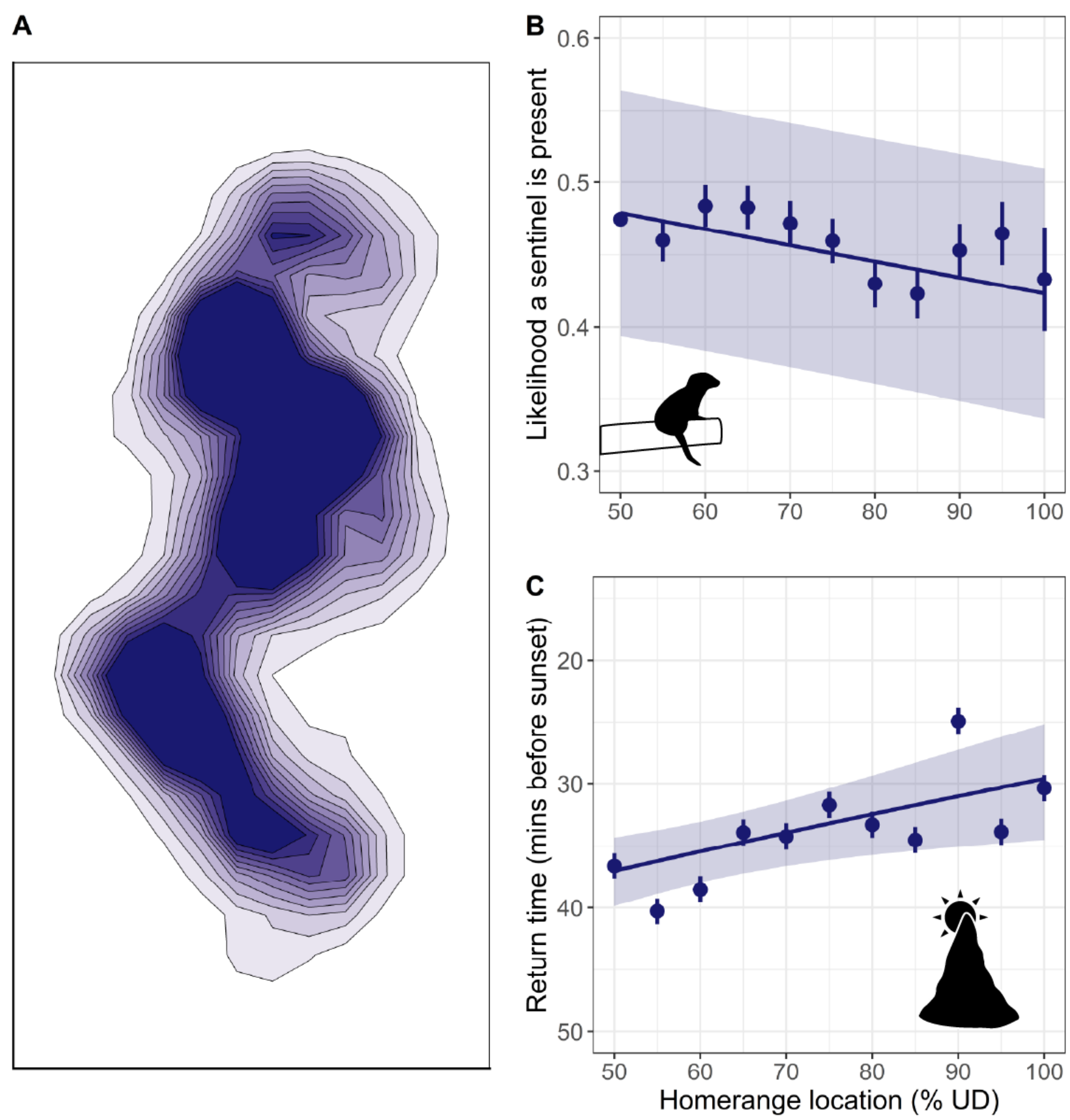

Supplementary Figure 5. Output of models investigating the relationship between homerange location and behaviour. (A) Layered utilisation distributions (UDs) of a mongoose group for a 90-day block, from 50% (dark) up to 95% (light) in 5% increments. Colour gradient corresponds with the x-axis of panels B-C; white corresponds to the area outside of the 95% UD (i.e., 100% UD). (B) The likelihood that a sentinel was present in a given scan-sample (N=22244 scan-samples). (C) Return time to the burrow in minutes before sunset, such that higher on the y-axis is later/closer to sunset (N=2567 evenings). (B-C) Lines and shading are model estimated marginal means and 95% confidence intervals. Points and arms are illustratively discretised raw data means and standard error.

Supplementary Table 9. Output of models investigating characteristics of sentinel behaviour relative to the location within the group's homerange: (A) likelihood that a sentinel was present, (B) sentinel bout duration and (C) likelihood that sentinel vocalised to announce presence. (A,C) Analysed as Generalised Linear Mixed Models with a binomial error structure and logit link function, (B) analysed as Linear Mixed Models with log-transformed response data. Confidence intervals (CI) are estimated as 95% from 1000 bootstraps, random term standard deviation italicised, significant fixed factors in bold. (A) N=22244 scan-samples across 4519 observation sessions with 12 groups, (B) N=13278 sentinel bouts across 3288 observation sessions with 12 groups, (C) N=12490 sentinel bouts across 3191 observation sessions with 12 groups.

Factor	Estimate	95% CI	χ^2	DoF	P
(A) Sentinel Presence					
Intercept	-0.015	-0.391, 0.372			
Absolute Group-Size	0.316	0.006, 0.602	3.583	1	0.058
Homerange Location	-0.069	-0.109, -0.030	6.789	1	**0.009**
Observation Session	*0.832*	*0.722, 0.823*			
Group ID $_{Intercept}$	*0.618*	*0.292, 0.863*			
Group ID $_{Absolute\ Group\ Size}$	*0.493*	*0.230, 0.671*			
Group ID $_{Location}$	*0.026*	*0.000, 0.066*			
(B) Sentinel Bout Duration					
Intercept	4.254	4.186, 4.327			
Absolute Group-Size	-0.073	-0.143, 0.001	3.109	1	0.078
Homerange Location	-0.054	-0.078, -0.026	10.234	1	**0.001**
Observation Session	*0.454*	*0.427, 0.479*			
Group ID $_{Intercept}$	*0.084*	*0.015, 0.136*			
Group ID $_{Absolute\ Group\ Size}$	*0.109*	*0.027, 0.176*			
Group ID $_{Location}$	*0.023*	*0.000, 0.050*			
(C) Sentinel Vocalisation					
Intercept	0.472	0.053, 0.848			
Absolute Group-Size	0.341	0.088, 0.604	4.910	1	**0.027**
Homerange Location	0.034	-0.030, 0.098	1.060	1	0.304
log(Bout Duration)	0.514	0.459, 0.556	466.330	1	**<0.001**
Observation Session	*1.117*	*0.948, 1.103*			
Group ID $_{Intercept}$	*0.605*	*0.027, 0.822*			
Group ID $_{Absolute\ Group\ Size}$	*0.361*	*0.101, 0.547*			
Group ID $_{Location}$	*0.056*	*$9.656e^{-7}$, 0.109*			

Supplementary Table 10. Output of Linear Mixed Models with log-transformed response data on return time to the evening sleeping burrow relative to location within the homerange. A constant was applied to all response values before transformation to remove negative values. Confidence intervals (CI) are estimated as 95% from 1000 bootstraps, random term standard deviation italicised, significant fixed factors in bold. N=2567 returns to evening burrows across 12 groups.

Factor	Estimate	95% CI	χ^2	DoF	P
Intercept	3.970	3.928, 4.015			
Absolute Group-Size	-0.042	-0.071, -0.015	5.936	1	**0.015**
Homerange Location	-0.047	-0.078, -0.016	6.901	1	**0.009**
Group ID $_{Intercept}$	*0.064*	*0.004, 0.105*			
Group ID $_{Absolute\ Group\ Size}$	*0.027*	*0.000, 0.054*			
Group ID $_{Location}$	*0.039*	*$1.352e^{-7}$, 0.068*			
