# Supplementary material for: Dwarf mongooses pre-emptively alter their behaviour relative to the threat posed by different rival groups
Source: Nat Ecol Evol. 2026 Jun 16;10(8):1521–30. doi: 10.1038/s41559-026-03104-3 (PMC13441901; doi:10.1038/s41559-026-03104-3)
Supplement: Supplementary file 2 [file 41559_2026_3104_MOESM2_ESM.pdf]

nature portfolio

Corresponding author(s): Joshua J Arbon

Last updated by author(s): Apr 20, 2026

Reporting Summary

Nature Portfolio wishes to improve the reproducibility of the work that we publish. This form provides structure for consistency and transparency in reporting. For further information on Nature Portfolio policies, see our Editorial Policies and the Editorial Policy Checklist.

Statistics

For all statistical analyses, confirm that the following items are present in the figure legend, table legend, main text, or Methods section.

n/a	Confirmed	
☐	☒	The exact sample size (*n*) for each experimental group/condition, given as a discrete number and unit of measurement
☐	☒	A statement on whether measurements were taken from distinct samples or whether the same sample was measured repeatedly
☐	☒	The statistical test(s) used AND whether they are one- or two-sided<br>*Only common tests should be described solely by name; describe more complex techniques in the Methods section.*
☐	☒	A description of all covariates tested
☐	☒	A description of any assumptions or corrections, such as tests of normality and adjustment for multiple comparisons
☐	☒	A full description of the statistical parameters including central tendency (e.g. means) or other basic estimates (e.g. regression coefficient) AND variation (e.g. standard deviation) or associated estimates of uncertainty (e.g. confidence intervals)
☐	☒	For null hypothesis testing, the test statistic (e.g. *F*, *t*, *r*) with confidence intervals, effect sizes, degrees of freedom and *P* value noted<br>*Give P values as exact values whenever suitable.*
☒	☐	For Bayesian analysis, information on the choice of priors and Markov chain Monte Carlo settings
☐	☒	For hierarchical and complex designs, identification of the appropriate level for tests and full reporting of outcomes
☒	☐	Estimates of effect sizes (e.g. Cohen's *d*, Pearson's *r*), indicating how they were calculated

Our web collection on statistics for biologists contains articles on many of the points above.

Software and code

Policy information about availability of computer code

Data collection	All data processing was conducted using R version 4.5.1, using the packages adehabitatHR (v0.4.22), sf (v1.0-24), MODISTools (v1.1.6).
Data analysis	All analyses were conducted using R version 4.5.1, using the packages lme4 (v1.1-38), glmmTMB (v1.1.14), car (v3.1-5), DHARMa (v0.0.7).

For manuscripts utilizing custom algorithms or software that are central to the research but not yet described in published literature, software must be made available to editors and reviewers. We strongly encourage code deposition in a community repository (e.g. GitHub). See the Nature Portfolio guidelines for submitting code & software for further information.

Data

Policy information about availability of data

All manuscripts must include a data availability statement. This statement should provide the following information, where applicable:

- Accession codes, unique identifiers, or web links for publicly available datasets
- A description of any restrictions on data availability
- For clinical datasets or third party data, please ensure that the statement adheres to our policy

All data supporting this submission can be accessed at doi.org/10.6084/m9.figshare.28574711

Research involving human participants, their data, or biological material

Policy information about studies with human participants or human data. See also policy information about sex, gender (identity/presentation), and sexual orientation and race, ethnicity and racism.

Reporting on sex and gender	N/A
Reporting on race, ethnicity, or other socially relevant groupings	N/A
Population characteristics	N/A
Recruitment	N/A
Ethics oversight	N/A

Note that full information on the approval of the study protocol must also be provided in the manuscript.

Field-specific reporting

Please select the one below that is the best fit for your research. If you are not sure, read the appropriate sections before making your selection.

☐ Life sciences ☐ Behavioural & social sciences ☒ Ecological, evolutionary & environmental sciences

For a reference copy of the document with all sections, see nature.com/documents/nr-reporting-summary-flat.pdf

Ecological, evolutionary & environmental sciences study design

All studies must disclose on these points even when the disclosure is negative.

Study description	This study investigates the behaviour of wild dwarf mongoose groups relative to the group size of their neighbours and their location within their homerange. This study uses 10 years of GPS, life history and behavioural data to investigate multiple behavioural metrics in a spatially explicit setting. Group behaviour was analysed both relative to the size of a neighbour group when the group was in a neighbour zone (within the homerange of the neighbour) as a proxy for a time when inter-group interactions are more common. Behaviour was also analysed relative to the location within each group's homerange as a different measure of inter-group threat level.
Research sample	A local population of up to 8 wild, habituated groups of dwarf mongooses (Helogale parvula). Family groups contained all ages from breeding adults to pups. For the purposes of these analyses, only individuals over 1 year old were considered.
Sampling strategy	All data collected during the long-term data collection of the Dwarf Mongoose Research Project were used to maximise statistical power.
Data collection	Data were collected by >50 trained observers who recorded GPS data using handheld devices, and noted behavioural observations into field notebooks that were then transferred to an on-site database.
Timing and spatial scale	Data were collected between September 2013 and September 2023. Groups of habituated mongooses were observed for 2-3 days per week over the period.
Data exclusions	Outlier data (those points falling more than 3*SD from the mean) were excluded to help achieve parsimonious model fit - these are reported in the methods.
Reproducibility	As this is a study on a 10-year observational data sample, no repeats were possible.
Randomization	As this is observational data from wild groups, there was no randomization process.
Blinding	All analyses presented were conceived after the end of the data collection period, therefore all observers were blind to the hypotheses we test

Did the study involve field work? ☒ Yes ☐ No

Field work, collection and transport

Field conditions	All fieldwork was conducted in a bushveld habitat within the Savanna biome in South Africa. The area is seasonal, with a hot, wet, summer period (Sep-Mar) and a cooler, drier winter period (Apr-Aug). All fieldwork was conducted on foot.

Location	All data were collected at Sorabi Rock Lodge, Limpopo Province, South Africa (24.211 S, 30.775 E).
Access & import/export	Work was conducted under permission from the Limpopo Department of Economic Development, Environment and Tourism (permit number: 001CPM403-00013), and ethical approval from the University of Pretoria, South Africa (Animal Ethics Committee: NAS321/2022) and the University of Bristol, UK (Animal Welfare and Ethics Review Body: UB/11/038), and in line with ASAB guidelines for the ethical treatment of animals.
Disturbance	Observations were conducted by trained researchers to minimise disturbace to the wild animals. All groups were habituated to close human presence and could be comfortably followed at <5 m.

Reporting for specific materials, systems and methods

We require information from authors about some types of materials, experimental systems and methods used in many studies. Here, indicate whether each material, system or method listed is relevant to your study. If you are not sure if a list item applies to your research, read the appropriate section before selecting a response.

Materials & experimental systems

n/a	Involved in the study	
☒	☐	Antibodies
☒	☐	Eukaryotic cell lines
☒	☐	Palaeontology and archaeology
☐	☒	Animals and other organisms
☒	☐	Clinical data
☒	☐	Dual use research of concern
☒	☐	Plants

Methods

n/a	Involved in the study	
☒	☐	ChIP-seq
☒	☐	Flow cytometry
☒	☐	MRI-based neuroimaging

Animals and other research organisms

Policy information about studies involving animals; ARRIVE guidelines recommended for reporting animal research, and Sex and Gender in Research

Laboratory animals	The study did not involve laboratory animals.
Wild animals	The study reports observations of 740 wild dwarf mongooses of all ages (Helogale parvula) over a 10-year period. All individuals were wild and habituated to the close presence of observers; no animal was ever trapped, tagged or implanted. Individuals were rendered uniquely identifiable by the application of blond hair dye to their fur with an elongated paintbrush.
Reporting on sex	All results reported are the aggregation of observations to the group level. As no individual-level data are reported, it is not possible to consider individual sex effects.
Field-collected samples	The study did not involve collecting samples from the field.
Ethics oversight	Work was conducted under permission from the Limpopo Department of Economic Development, Environment and Tourism (permit number: 001CPM403-00013), and ethical approval from the University of Pretoria, South Africa (Animal Ethics Committee: NAS321/2022) and the University of Bristol, UK (Animal Welfare and Ethics Review Body: UB/11/038), and in line with ASAB guidelines for the ethical treatment of animals.

Note that full information on the approval of the study protocol must also be provided in the manuscript.

Plants

Seed stocks	*Report on the source of all seed stocks or other plant material used. If applicable, state the seed stock centre and catalogue number. If plant specimens were collected from the field, describe the collection location, date and sampling procedures.*
Novel plant genotypes	*Describe the methods by which all novel plant genotypes were produced. This includes those generated by transgenic approaches, gene editing, chemical/radiation-based mutagenesis and hybridization. For transgenic lines, describe the transformation method, the number of independent lines analyzed and the generation upon which experiments were performed. For gene-edited lines, describe the editor used, the endogenous sequence targeted for editing, the targeting guide RNA sequence (if applicable) and how the editor was applied.*
Authentication	*Describe any authentication procedures for each seed stock used or novel genotype generated. Describe any experiments used to assess the effect of a mutation and, where applicable, how potential secondary effects (e.g. second site T-DNA insertions, mosiacism, off-target gene editing) were examined.*
